# Yoga and pain: A mind-body complex system

**DOI:** 10.3389/fpain.2023.1075866

**Published:** 2023-02-23

**Authors:** Deepak Chopra, Eddie Stern, William C. Bushell, Ryan D. Castle

**Affiliations:** ^1^ The Chopra Foundation, New York, NY, United States; ^2^ Vivekananda Yoga University, Los Angeles, CA, United States; ^3^ Chopra Foundation Institute, New York, NY, United States; ^4^ Chopra Foundation Institute, Honolulu, HI, United States

**Keywords:** pain, yoga, pain managemant, complex adaptive systems, interoception, systems theory, mind - body

## Abstract

**Introduction:**

The human body's response to pain is indicative of a complex adaptive system. Therapeutic yoga potentially represents a similar complex adaptive system that could interact with the pain response system with unique benefits.

**Objectives:**

To determine the viability of yoga as a therapy for pain and whether pain responses and/or yoga practice should be considered complex adaptive systems.

**Methods:**

Examination through 3 different approaches, including a narrative overview of the evidence on pain responses, yoga, and complex system, followed by a network analysis of associated keywords, followed by a mapping of the functional components of complex systems, pain response, and yoga.

**Results:**

The narrative overview provided extensive evidence of the unique efficacy of yoga as a pain therapy, as well as articulating the relevance of applying complex systems perspectives to pain and yoga interventions. The network analysis demonstrated patterns connecting pain and yoga, while complex systems topics were the most extensively connected to the studies as a whole.

**Conclusion:**

All three approaches support considering yoga a complex adaptive system that exhibits unique benefits as a pain management system. These findings have implications for treating chronic, pervasive pain with behavioral medicine as a systemic intervention. Approaching yoga as complex system suggests the need for research of mind-body topics that focuses on long-term systemic changes rather than short-term isolated effects.

## 1. Introduction

The human body's response to pain is indicative of a complex adaptive system. While some pain interventions are simple and mechanical, others are themselves complex systems capable of interfacing and influencing the body's nervous system. Therapeutic yoga, in addition to other forms of mind-body therapy, potentially represents such a complex adaptive system. Its multimodal approach produces results expected from a complex adaptive system, more so than could be expected from reducing therapeutic yoga to simple calisthenics. Making this determination is valuable for determining the efficacy and range of benefits related to therapeutic yoga, as complex systems cannot be thoroughly modeled with reductionist methodologies. Without understanding the systemic effect yoga can have on pain, optimal treatment plans will remain incomplete and susceptible to overuse of temporary analgesics such as opioids.

## 2. Background

Yoga has long been used as a treatment for pain, especially chronic pain, and the interactions between pain responses and yoga practice are well documented. Many of the interactions between yoga practice and pain responses demonstrate behavior common to complex systems, which could necessitate new methodologies to study pain management. As current research has not confirmed the presence of such a system, an examination of the basic mechanisms behind pain responses, yoga as a pain treatment, and complex systems is necessary in order to identify whether it is likely a complex system may be present.

### 2.1. Pain

Pain is an informative sense perception of the brain, an interpretation of signals sent from the limbs, muscles, and organs *via* afferent nerves of the peripheral nervous system, as a protective modality. In contrast to other information-based organs such as the eyes, ears, etc., the sense of pain serves as a deterrent in order to prevent more pain, avoid dangerous situations, and provide diagnostic information about which unseen part of the body (under the skin) might be the cause of pain (1). There are no pain nerves as such, just signals that the brain determines should be tagged as painful (2, 3). Pain is termed nociceptive, the sensory receptors that detect signals from damaged tissues are called nociceptors, and the central nervous system's process of interpreting pain signals is called nociception. There are different types of nociceptors that convey thermal, mechanical, and chemical messages, and silent nociceptors that become responsive during periods of inflammation.

The sensory feedback system of pain responses is a critical component of pain management and one of the main areas of potential interaction with the practice of yoga.

### 2.2. Yoga

Though sometimes reduced to an exercise program, yoga is a multi-modality practice dating to at least 2700 BCE and which encompasses a variety of components. Traditional yoga observances include postures, breathing, behavior, meditation, and devotional practices that primarily serve the philosophical undertaking of self-knowledge or spiritual liberation (4). In ancient India, dating to approximately 1st to 2nd century BCE, yoga was also described as a practice that steadies the sense organs (5).

**Figure 1:**
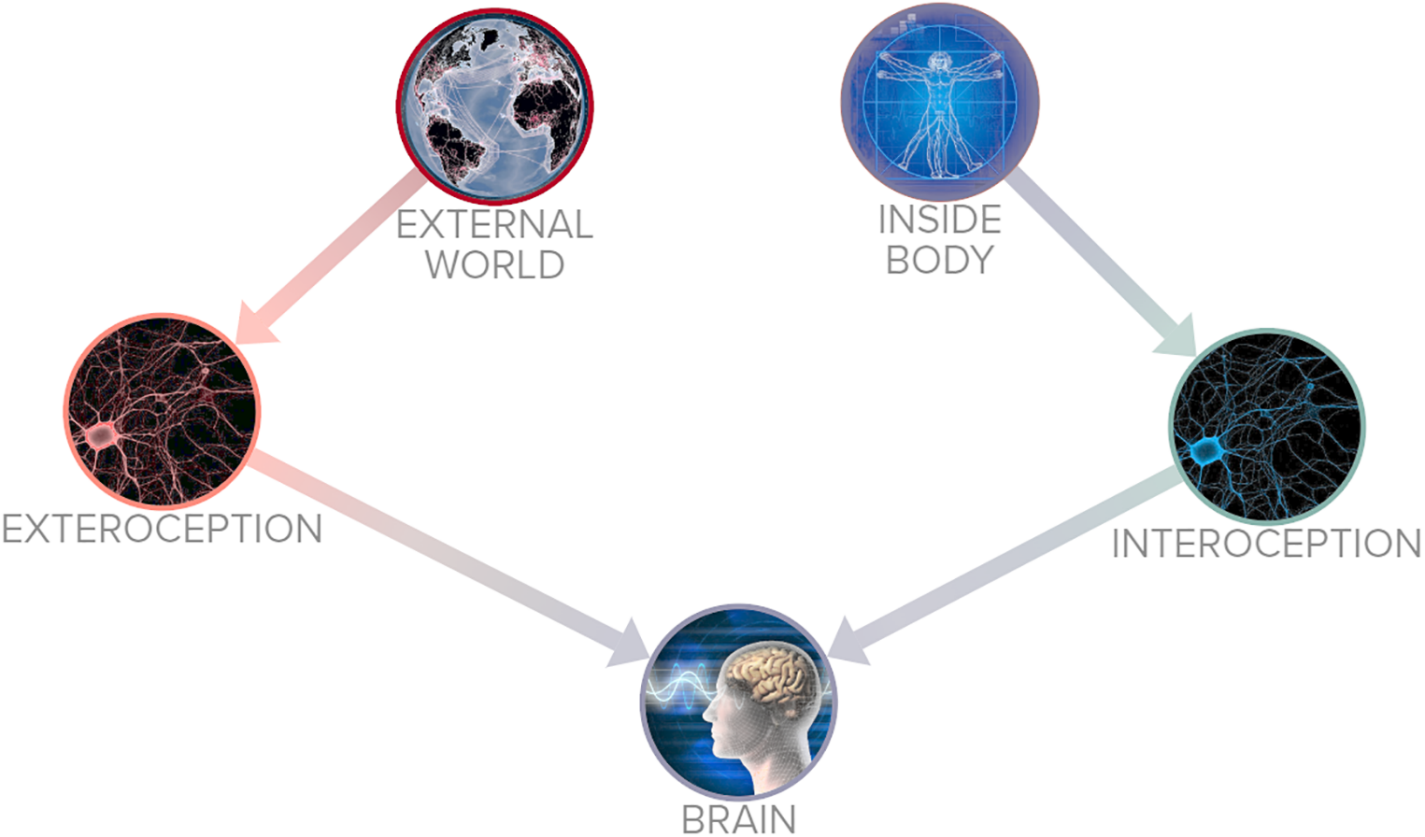
Interoceptive and exteroceptive perception.

Yoga's relationship with sensory perception is relevant to its use in pain management. The sense organs are pathways of perception that convey incoming information from the environment to the central nervous system (6). In the human body there are sensory nerve fibers that convey information to the central nervous system, often divided in external perceptions (exteroception): detecting pressure, pain, change in temperature, and internal perceptions (interoception): the need for food, sleep, and evacuation of the bowels and bladder (3, 7). Figure 1 outlines the basic function of interoception and exteroception. In traditional yoga systems the two sensory pathways of exteroception and interoception are not mutually exclusive (8).

11th to 17th century texts (*Hatha Yoga Pradipika*, *Gheranda Samhita*, *Yoga Hatharatnavali*, *Siva Samhita*) describe the practice of yoga for strength-training, flexibility, bolstering the immune system, and improving longevity (9). Contemporary yoga practices have been adapted into the cultural milieu of almost every country in the world, postures (*asanas*) having become the primary indicator or representation of yoga practice (10). In addition to cultural purposes, the modern practice of yoga has a growing body of evidence-based research showing efficacy as an adjunctive mind-body therapy and is increasingly used to improve healthcare outcomes (11–14).

Evidence supports the benefits of yoga for numerous health problems, including:

• stress-reduction and improving debilitating pain (15, 16)
• orthopedic problems such as back pain, knee pain, or other musculoskeletal illnesses (17, 18)
• stomach pain (19)
• psychological illnesses (20–22)
• sleep disruption (23)
• cardiovascular disease (24–26)
• diabetes (27)
• inflammatory disorders (28, 29)
• immunological illnesses (30)

Yoga's impact on pain is especially well documented and suitable for further examination given the thorough documentation of pain in patients and the reliability its associated biomarkers across the human system (31). The combination of interactions between pain responses and yoga practice create the potential need for a complex systems approach.

### 2.3. Complex systems

Complex systems are holistic phenomena that cannot be easily modeled through standard reductionist methods. The study of complex systems focuses on relationships between networks within a system and the sometimes unpredictable behavior that emerges when those relationships change (32). Complex systems are not limited to any scholarly discipline and can occur in physics as readily as in neuroscience or economics. Examples include ant nests, climate events, the healthcare industry, or the immune system (33). Scale is less relevant to a system than the strength of the relationships between its network of parts, or nodes (34). Complex systems are often composted of other complex systems, like the economy being comprised of companies, being staffed by humans, all of which are complex systems.

The characteristics of a complex system are important to understanding the study of a system:

• Emergent: its behavior is an emergent phenomenon that operates holistically. The behavior of the system as a whole cannot be reliably extrapolated from studying its parts in isolation, much like the ocean tides cannot be modeled from a drop of water.
• Dynamic networks: interdependent means of communicating reactions between nodes are a critical part of the complexity of a system (35). This network involves both unpredictable stochastic dynamics and multiple scales of simple interactions. Dropping a rock in a pond will send ripples through the pond’s interdependent parts, while dropping a rock on the sand beside the pond would have no such impact because it is outside the dynamic network.
• Feedback loops: both damping and amplifying feedback cycles are found in complex systems, such that changes within the system can cause further, cyclical changes. This requires detectors to recognize input and agents to adjust behavior.
• Open system: complex systems are generally energy-rate-dense and far from energetic equilibrium. To sustain cohesion they must take in energy as they expend energy overall, though they may maintain systemic stability (36).
• Nonlinear: the effects of changes to a complex system are not necessarily proportional to the size of the change. Removing 10% of a human’s body does not leave the human 90% functional.

A deeper component involves the adaptive capability of the system in question. Complex adaptive systems (CAS) are capable of changing in response to stimuli while continuing to self-organize (37). Many biological or health systems are complex adaptive systems and cannot be fully understood unless approached as such (38).

Another method of determining systemic influence is by performing a network analysis. Network analyses quantify the strength of relationships within a connected system, revealing components that share various behaviors or qualities. Networks that show very weak or inconsistent connections across a network analysis are unlikely to represent a complex system.

The identification and study of complex systems is a valuable scientific process, especially within the fields of health and wellbeing (39). If researchers expect their subject to demonstrate an isolated effect when it is actually a complex system, they will not be measuring the full range of interdependent ripple effects from their tests. If interventions are expected to produce a simple, linear reaction within one network, they will be unable to predict or explain a disproportionate reaction if the system is complex. Most importantly, attempts to explain a complex adaptive system's behavior by reviewing components in isolation will overlook the emergent properties of the system's behavior, producing outcomes that seem unpredictable or contradictory.

Health outcomes and interventions cannot be optimized without awareness and study of the complex adaptive systems being engaged (40).

## 3. Methodology

This paper has multiple aims culminating in a comprehensive assessment of the existence and/or relevance of yoga’s interactions with pain as a complex adaptive system.

1. Evidence overview: a narrative summary of the evidence behind therapeutic yoga for pain. This will determine the efficacy of yogic interventions and which aspects of yoga have been reliably associated with pain responses.
2. Literature review and network analysis: a collection of existing publications on the various aspects of this topic will be collated and relevant keywords isolated. Studies and keywords will be examined through network analysis to discover any patterns that could be used to determine the presence of a connected system.
3. Complex adaptive system mapping: a visual map of the basic framework common to complex adaptive systems will be cross-referenced with mappings of pain responses and yoga’s interactions with pain pathways. This will help determine whether pain and yoga’s effects on it resemble a CAS.

### 3.1. Evidence overview

This section will consist of a contextualized narrative of the evidence, as outlined by experts in yoga, chronic illness, mind-body interventions, and systems theory. As a summary this section will focus on clarity and qualifying explanations rather than isolated metrics.

### 3.2. Literature review

**Figure 2:**
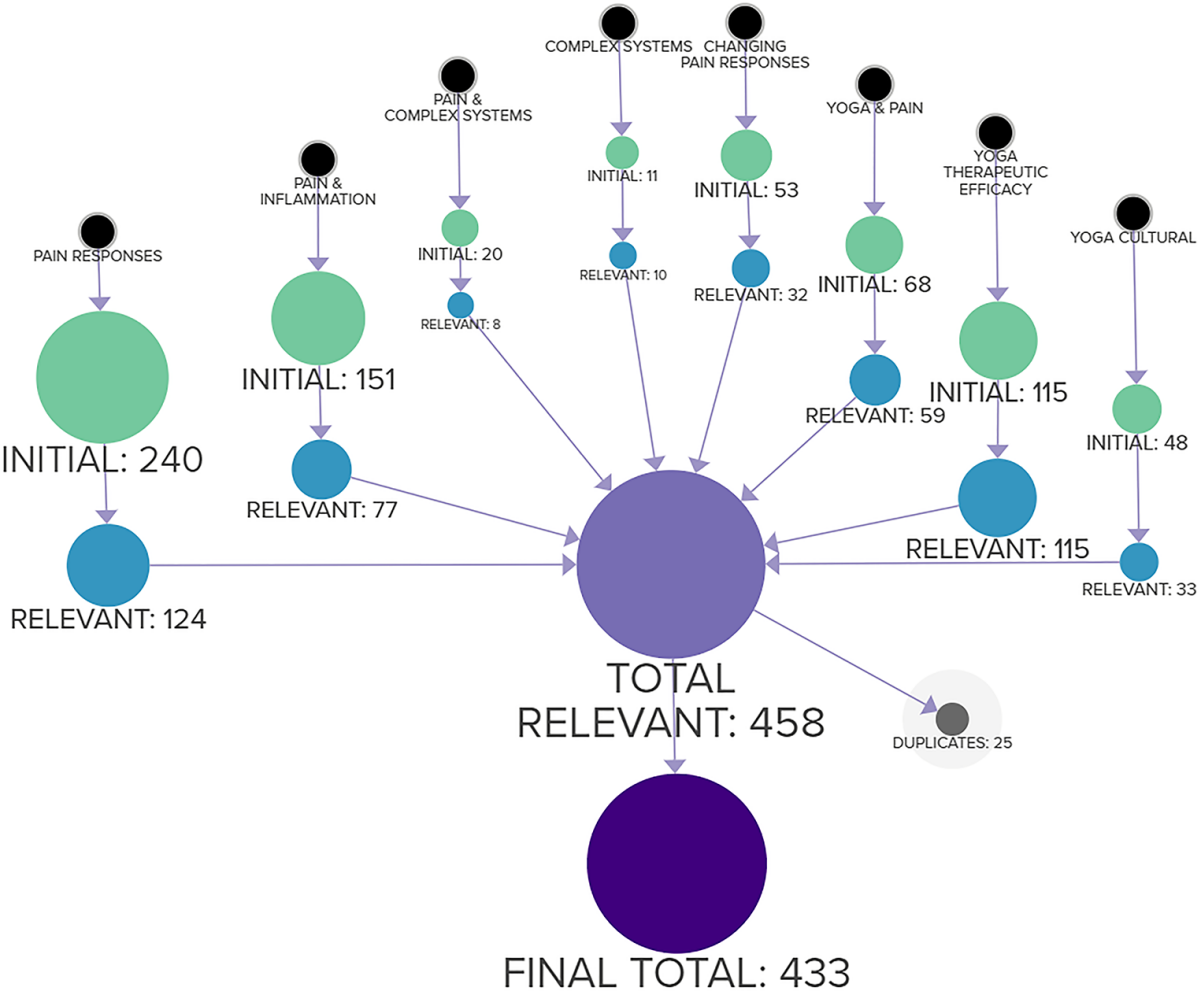
Methodology for selection of literature, beginning with individual topics and ending with final total to be reviewed.

The search terms below were used to capture studies relevant to this paper. Reasons for exclusion include being irrelevant to topic, hyperspecific (dealing exclusively with singular conditions/demographics), overly technical (genetic profiles, fMRI calibration, etc), or overly broad (hypotheticals, unverified predictions, speculation). Search terms were designed to identify factors relevant to this review, such as modes of perception or cultural/pain management components of yoga, in order to prevent overly generalized results. Search filters required the inclusion of abstracts and only studies related to humans. Databases searched include PubMed and arXiv.org. Figure 2 provides a diagram of the search and filtering process.

### 3.3. Network analysis

There were 706 initial results, after reviewing titles and abstracts this was reduced to 458 studies relevant to the purpose of this study, after elimination of duplicates the final total was 433. The relevant studies were then processed through factor mapping software, identifying tags and keywords to calculate commonalities and confluences across studies.

**Figure 3:**
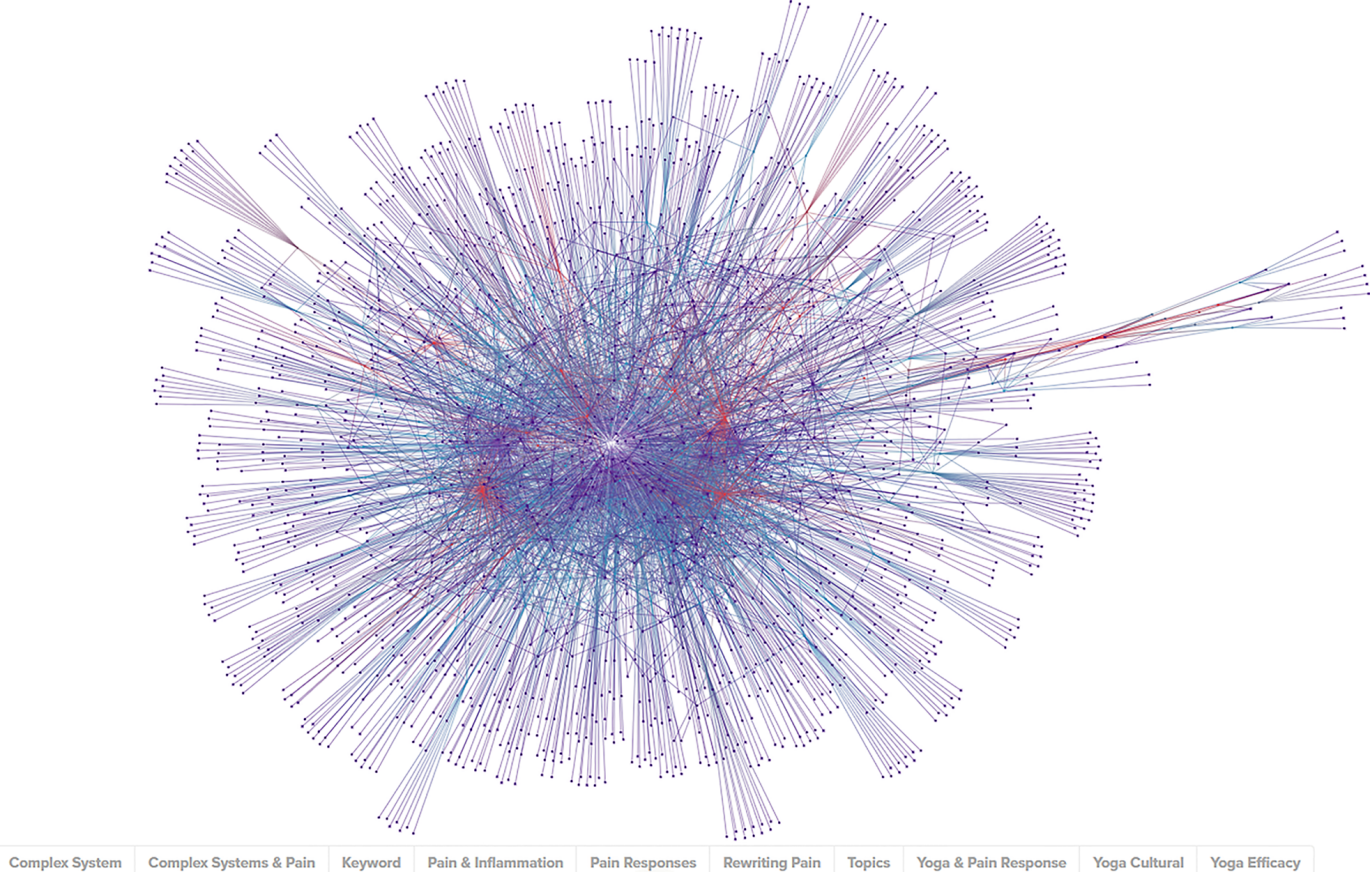
Graphic representation of nodes made up of KEYWORDS (purple), STUDIES (blue), and TOPICS (red) captured in the literature review.

The combined keywords across the 433 studies resulted in 1,639 unique tagged keywords. Connections, intersections, and frequency of use across all studies was calculated for each tagged keyword. The results were modeled spatially then subjected to network analysis, as seen in Figure 3.

The network analysis was performed to determine the studies most linked by these keywords and the results were sorted by the network metrics of degree, closeness, and eigenvector. Measured types of nodes include KEYWORD (content-specific tags relating to the subject of a study or studies), STUDY (peer-reviewed publications on the related topics), and TOPIC (the overarching subject matter behind the literature review search). The network and analytics are available online (Supplementary Material).

### 3.4. Functional systems mapping

In order to identify whether yoga's interactions with pain constitute a complex adaptive system, a diagram of the essential functions required of a CAS was designed. Yoga and pain interactions will be overlaid onto relevant parts of the diagram to see if the systems correspond. A further overlay will examine the broader application of mind-body therapies for the same purpose.

## 4. Results

### 4.1. Evidence overview

#### 4.1.1. Pain pathways

Nerves carry messages regarding pain from the body through the peripheral nervous system to interneurons that pass that information to the brain. Interneurons can inhibit or speed up the passing of information through ion channels, which are temporary openings in the nerves that respond to different stimuli and make the nerves “fire” (3). The brain responds in three basic ways to stimuli:

• Peripheral sensitization, creating more inflammation for healing
• Central sensitization, increasing sensitivity between the nerve from the injured area and the nerves to the brain
• Cortical reorganization, the part of the brain that maps to the injured body part becomes bigger, ie. mirror therapy

Chronic pain causes the central nervous system to become more sensitive and signals increase accordingly, beginning a detrimental feedback loop. The brain maps sensations as information by tagging it according to neurological purposes (neuro-tags). The brain stem receives signals and interprets, associating the tagged information with higher centers related to emotions, memory, and perception. Though each individual's sensation map and pain perception differ, the system that creates those maps and perceptions are alike.

The perceptions tagged onto signals can make a significant difference in health outcomes following prolonged or chronic pain. Catastrophizing belief patterns such as “pain is terrifying,” or, “I’ll never recover from this or regain my earlier capabilities,” and other emotional issues contribute to increased levels of pain perception, which in turn increases the feedback loop of pain severity (41). There is evidence that educating patients about the relationship between their brain, body, and pathways of pain leads to greater recovery, especially when combined with therapeutic exercises (42–44). Without education on pain, and especially for those that have low thresholds for pain sensitivity, catastrophizing can lead to slower recovery rates and greater levels of pain interpretation. Poor body awareness presents with higher levels of pain sensitivity, indicating that mind-body modalities like yoga that encourage positive body awareness and heightened levels of interoception are helpful adjunctive treatments for chronic pain (45, 46).

#### 4.1.2. Yoga and pain

Yoga is a multi-modality practice that uses both top-down and bottom-up interactions between the central nervous system and peripheral nervous system. These bidirectional interactions have an impact on physiological and emotional health, in part by controlling peripheral inflammatory responses that are involved in pain signaling (47). Yoga is known through multiple studies to down-regulate sympathetic nervous system hyper-arousal through the HPA axis, increase parasympathetic activation through the vagal nerve complex, and reduce allostatic load to help stabilize the autonomic nervous system (46). Improvement of vagal tone is closely linked to yoga and helps control the inflammatory response of the adrenergic reactions to chronic anxiety, providing potential mitigation of the detrimental feedback loop of stress and pain (2, 48).

Practice of yoga also leads to a decrease in pain perception through cognitive disengagement, leading to a decrease in the affective aspect of pain sensitivity and increasing interoception (46). Pain tolerance is increased and the anxiety associated with pain are decreased through reducing the hyper-arousal of the HPA axis and thus reducing the output of stress hormones. This occurs through interaction with the brain structures that support the perception of stress and support mood and cognitive abilities, including the amygdala, insula, and hippocampus (48).

The movement of yoga provides pain benefits beyond basic calisthenics. Through gentle and safe movements, the relief from fear of movement being “bad” can be alleviated (46). Yoga enhances positive body awareness through both interoception and proprioceptive integration, reducing the anticipation of pain and thereby reducing the sensitization to it. The catastrophizing that accompanies pain and the hesitancy to move can be alleviated, further reducing pain feedback loops. Gentle, guided movement also improves the over sensitization of the nervous system that can occur due to pain (41).

When the sympathetic-parasympathetic balance is restored due to yoga and other mind-body practices, new neuro-tags are created which allow for a reframing of past experiences (49). Prolonged HPA activation leads to cognitive deficits due to high levels of cortisol, but yoga helps to downregulate the stress response and prevent this deterioration.

Movement is a critical part of yoga, but the effective yoga practice involves other mind-body components, especially meditation (50). Mindfulness meditation is known to be helpful for reducing anticipatory thoughts, an important step in the sensitization process (51). There is evidence that the anticipation of pain create neural response that neuro-tags information for pain perception, influencing entire networks of attention and stimuli response to anticipate pain (52). A study on 160 chronic pain patients showed that greater acceptance of pain led to lower levels of reported pain perception, less pain anxiety, depression, and associated problems of chronic pain, all independent of pain intensity (53). Yoga and meditative practices have a firm grounding in acceptance, surrender, and relaxation, which can contribute to cognitive reframing of the catastrophizing most strongly associated with perceived pain intensity (54).

As a multi-modality practice, yoga consists of postures, breathing practices, meditation, and attention to diet and sleep habits. Lifestyle habits can have systemic impacts on the neuro-tags attached to signals from the peripheral nervous system, as well as the neural structure itself (45). A study on experienced yoga practitioners with matched controls showed more gray matter in multiple brain regions of yoga practitioners. An important region impacted included the insula, which is closely correlated with pain tolerance. Yoga practitioners also exhibited increased intra-insular white matter consistent with nociceptive input and parasympathetic regulation. These structural improvements in combination with cognitive reframing and improved interoceptive awareness suggest a significant influence on the system of pain perception (55).

#### 4.1.3. Complex systems of mind-body interactions

The pain response system, especially in relation to chronic pain, is considered typical of a complex adaptive system (56). All of the required components are present in the nociceptive network, the body's behavior in response to pain is based on detectors and signaling agents, and the unpredictable nature of pain's long-term feedback loops are nonlinear emergent phenomena. The factors associated with the mind-body response to pain are widely studied as a complex system on multiple scales (57).

Approaching mind-body therapies for pain as CAS is much rarer and an overdue field of study. The CAS similarity between the mind-body response to pain and mind-body practices like yoga or meditation have been explored and suggested, but never quantified or outlined (58). The simplest and clearest evidence that mind-body therapies are a CAS lies in the fact that mind-body therapies like yoga are capable of interacting with pain on a systemic level (59). If pain responses are a complex adaptive system, and yoga has been shown to interface with the body's responses through similar methods and scales, logically yoga should be considered a CAS.

Practical concerns and public health utility require more detailed justification, however. A comparison of the mind-body response to both pain and yoga with the components of a complex adaptive system is warranted.

• **Emergent**: through systemic interactions pain can produce behavior in the nervous system that cannot be easily reduced or isolated (60). Yoga likewise involves exercises, breathing practices, and mental states that affect a wide number of agents on a systemic level (61). Evidence suggests that isolated physical components of yoga can benefit specific conditions like hypertension, but combinations of breathing, meditation, and mental practices produce a wide range of physical and mental health benefits (15).
• **Dynamic networks**: pain responses are communicated through the peripheral nervous network, HPA axis, and intricate series of interactions between large and small networks of nociceptive responses (62). Mind-body therapies engage physiological networks associated with exercise, psychological networks associated with stress, and neurological networks associated with neuro-tagging pain signals (46). Both of these sets of relationships represent dynamic networks consisting of stochastic interactions and multi-scalar systems.
• **Feedback loops**: both pain and mind-body therapies demonstrate the ability to initiate and modulate feedback loops in humans. An example of a feedback loop influenced by both chronic pain and yoga is the inflammatory cycle (63).
• **Open system**: as with nearly any biological function, both pain responses and mind-body practices are far from energetic equilibrium, requiring significant caloric intake and expending energy as either physical movement or neurological reaction (64).
• **Nonlinear**: the effects of chronic pain are often dramatically disproportionate to the ongoing harm stimuli the body is experiencing, constituting a nonlinear reaction (65). Mind-body interventions like yoga have likewise demonstrated improvements across physical, cognitive, and psychological health that are disproportionate to the amount of time spent practicing (66).

### 4.2. Network analysis results

Table 1 outlines the search terms included in the network analysis and the bodies of results used to determine which articles and keywords to track.

**Table 1:** List of search terms, results, and filtering for relevancy sorted by category.

**1. Topic: Pain responses**
1.1. Search terms: (interocept*[Title/Abstract]) AND (nocicept*[Title/Abstract]); (exterocept*[Title/Abstract]) AND (nocicept*[Title/Abstract]); (sensitization*[Title/Abstract]) AND (catastrophiz*[Title/Abstract]);
1.2. Number of initial results: 240
1.3. Number of relevant results after perusal: 124
**2. Topic: Pain response and inflammation**
2.1. Search terms: (pain sensitization[Title/Abstract]) AND (inflamm*[Title/Abstract]); (catastrophiz*[Title/Abstract]) AND (inflamm*[Title/Abstract])
2.2. Number of initial results: 151
2.3. Number of relevant results after perusal: 77
**3. Topic: Yoga cultural background**
3.1. Search terms: *(yoga[Title/Abstract]) AND (cultural[Title/Abstract])*
3.2. Number of initial results: 48
3.3. Number of relevant results after perusal: 33
**4. Topic: Yoga and pain**
4.1. Search terms: (yoga[Title/Abstract]) AND (nocicept*[Title/Abstract]); (yoga[Title/Abstract]) AND (interocept*[Title/Abstract]); (yoga[Title/Abstract]) AND (pain catastroph*[Title/Abstract]); (yoga[Title/Abstract]) AND (cytokine*[Title/Abstract])
4.2. Number of initial results: 68
4.3. Number of relevant results after perusal: 59
**5. Topic: Changing pain responses**
5.1. Search terms: (cortical reorganization[Title/Abstract]) AND (behavior[Title/Abstract]); (pain education[Title/Abstract]) AND (inflamm*[Title/Abstract]); (pain educat*[Title/Abstract]) AND (sensitiv*[Title/Abstract])
5.2. Number of initial results: 53
5.3. Number of relevant results after perusal: 32
**6. Topic: Complex systems**
6.1. Search terms: ARXIV (complex system[Title/Abstract]) AND (dynamic*[Title/Abstract])
6.2. Number of initial results: 11
6.3. Number of relevant results after perusal: 10
**7. Topic: Pain and complex systems**
7.1. Search terms: *PUBMED; ARXIV (pain[Title/Abstract]) AND (complex adaptive system[Title/Abstract])*
7.2. Number of initial results: 20
7.3. Number of relevant results after perusal: 8
**8. Topic: Yoga efficacy (Due to a large number of responses the following search term was limited to systematic reviews to improve efficiency and reduce disproportionate weighting of keywords)**
8.1. (yoga[Title/Abstract]) AND (efficacy*[Title/Abstract])
8.2. Number of initial results: 115
8.3. Number of relevant results after perusal: 115

#### 4.2.1. Betweenness

Betweenness is a measure of centrality in a graph based on shortest paths. For every pair of node in a connected network, there exists at least one shortest path between the vertices such that the number of edges that the path passes through (67). The betweenness for each node is the number of these shortest paths that pass through the node. Betweenness represents the degree to which nodes stand between each other. While other metrics identify nodes that have the greatest input or output, betweenness helps identify the most heavily trafficked pathways.

As seen in Table 2, the nodes with the highest betweenness tended to be STUDIES, with the highest values occurring in nodes about complex systems, followed closely by nodes related to pain pathways and pain management. There are a small number of nodes related to yoga or mind-body interventions No KEYWORDS were in the top 30.

**Table 2:** Tracking the studies or keywords that showed the highest value in this metric. The betweenness for each node is the number of the shortest connecting paths that pass through the node.

Label	Type	Metric	Value
Small Open Chemical Systems Theory and Its Implications to Darwinian Evolutionary Dynamics, Complex Self-Organization and Beyond	Study	betweenness	1.76E-05
Extreme value theory of evolving phenomena in complex dynamical systems: firing cascades in a model of neural network	Study	betweenness	1.66E-05
Understanding and Modelling the Complexity of the Immune System: Systems Biology for Integration and Dynamical Reconstruction of Lymphocyte Multi-Scale Dynamics	**Study**	betweenness	1.54E-05
Complexity of Model Testing for Dynamical Systems with Toric Steady States	**Study**	betweenness	1.5E-05
Designer dynamics through chaotic traps: Controlling complex behavior in driven nonlinear systems	**Study**	betweenness	7.55E-06
Life as Complex Systems—Viewpoint from Intra-Inter Dynamics	**Study**	betweenness	6.14E-06
Angiotensin II Triggers Peripheral Macrophage-to-Sensory Neuron Redox Crosstalk to Elicit Pain	**Study**	betweenness	5.35E-06
*Common Brain Mechanisms of Chronic Pain and Addiction*	**Study**	betweenness	4.44E-06
*Immediate preoperative outcomes of pain neuroscience education for patients undergoing total knee arthroplasty: A case series*	**Study**	betweenness	4.32E-06
Applying Complexity Theory to a Dynamical Process Model of the Development of Pathological Belief Systems	**Study**	betweenness	4.18E-06
The evolution of self-control	**Study**	betweenness	4E-06
*Low- Versus High-Intensity Plyometric Exercise During Rehabilitation After Anterior Cruciate Ligament Reconstruction*	**Study**	betweenness	3.95E-06
Forecasting transitions in systems with high dimensional stochastic complex dynamics: A Linear Stability Analysis of the Tangled Nature Model	**Study**	betweenness	3.58E-06
*A Mechanism-Based Approach to the Management of Osteoarthritis Pain*	**Study**	betweenness	3.53E-06
*Influence of a periodized circuit training protocol on intermuscular adipose tissue of patients with knee osteoarthritis: protocol for a randomized controlled trial*	**Study**	betweenness	3.33E-06
*Psychological processing in chronic pain: a neural systems approach*	**Study**	betweenness	3.21E-06
Corticotrophin-releasing factor 1 activation in the central amygdale and visceral hyperalgesia	**Study**	betweenness	3.2E-06
Cultural adaptation framework of social interventions in mental health: Evidence-based case studies from low- and middle-income countries	**Study**	betweenness	3.17E-06
Elite competitive swimmers exhibit higher motor cortical inhibition and superior sensorimotor skills in a water environment	**Study**	betweenness	3.13E-06
Remote ischemic conditioning as a cytoprotective strategy in vasculopathies during hyperhomocysteinemia: An emerging research perspective	**Study**	betweenness	3.12E-06
*Assessing for unique immunomodulatory and neuroplastic profiles of physical activity subtypes: a focus on psychiatric disorders*	**Study**	betweenness	3.12E-06
Impact of complex, partially nested clustering in a three-arm individually randomized group treatment trial: A case study with the wHOPE trial	**Study**	betweenness	3.06E-06
Changes of meningeal excitability mediated by corticotrigeminal networks: a link for the endogenous modulation of migraine pain	**Study**	betweenness	3.04E-06
A Dynamical Similarity Approach to the Foundations of Complexity and Coordination in Multiscale Systems	**Study**	betweenness	2.87E-06
Creating Inclusive Physical Activity Spaces: The Case of Body-Positive Yoga	**Study**	betweenness	2.86E-06
Using focus group methods to develop multicultural cancer pain education materials	**Study**	betweenness	2.77E-06
A Randomized, Single-Blind Study Evaluating the Effect of a Bone Pain Education Video on Reported Bone Pain in Patients with Breast Cancer Receiving Chemotherapy and Pegfilgrastim	**Study**	betweenness	2.75E-06
A dynamical model of fast cortical reorganization	**Study**	betweenness	2.74E-06
General Pathways of Pain Sensation and the Major Neurotransmitters Involved in Pain Regulation	**Study**	betweenness	2.74E-06
*Complex System*	**Topics**	betweenness	0.000119

#### 4.2.2. Closeness

The value of closeness centrality, or closeness, determines the distance each vertex is from every other vertex. Points with high closeness tend to be highly correlated with the trends of the broader network. In this study closeness reflects many of the same patterns as degree centrality with slight variations due to interpreting limited quality from its connections, meaning connection to other highly connected vertices increases the value. This indicates a highly interdependent part of the network.

As seen in Table 3, the nodes with the largest closeness values were TOPICS, especially related to pain pathways, yoga interventions, and inflammation. Complex systems ranked relatively low as a TOPIC. STUDIES were significantly lower in value and were led by nodes related to pain pathways, inflammation, sensitization/catastrophization, and mind-body treatments. No KEYWORDS were present in the top 30.

**Table 3:** Tracking the studies or keywords that showed the highest value in this metric. The value of closeness centrality, or closeness, determines the distance each vertex is from every other vertex.

Label	Type	Metric	Value
Angiotensin II Triggers Peripheral Macrophage-to-Sensory Neuron Redox Crosstalk to Elicit Pain	Study	closeness	0.01494
Psychological processing in chronic pain: a neural systems approach	Study	closeness	0.014458
Immediate preoperative outcomes of pain neuroscience education for patients undergoing total knee arthroplasty: A case series	**Study**	closeness	0.013976
*Influence of a periodized circuit training protocol on intermuscular adipose tissue of patients with knee osteoarthritis: protocol for a randomized controlled trial*	**Study**	closeness	0.013976
Biopsychosocial Influence on Shoulder Pain: Influence of Genetic and Psychological Combinations on Twelve-Month Postoperative Pain and Disability Outcomes	**Study**	closeness	0.013494
*Common Brain Mechanisms of Chronic Pain and Addiction*	**Study**	closeness	0.01253
*Assessing for unique immunomodulatory and neuroplastic profiles of physical activity subtypes: a focus on psychiatric disorders*	**Study**	closeness	0.012048
*Low- Versus High-Intensity Plyometric Exercise During Rehabilitation After Anterior Cruciate Ligament Reconstruction*	**Study**	closeness	0.012048
*A Mechanism-Based Approach to the Management of Osteoarthritis Pain*	**Study**	closeness	0.011566
Disease-Related, Nondisease-Related, and Situational Catastrophizing in Sickle Cell Disease and Its Relationship With Pain	**Study**	closeness	0.011566
Mind-body therapies and control of inflammatory biology: A descriptive review	**Study**	closeness	0.011566
Pain, psychosocial tests, pain sensitization and laparoscopic pelvic surgery	**Study**	closeness	0.011566
Pilot study of inflammatory responses following a negative imaginal focus in persons with chronic pain: analysis by sex/gender	**Study**	closeness	0.011566
Biopsychosocial influence on shoulder pain: Rationale and protocol for a pre-clinical trial	**Study**	closeness	0.011084
Generalized Pain Sensitization and Endogenous Oxytocin in Individuals With Symptoms of Migraine: A Cross-Sectional Study	**Study**	closeness	0.011084
Inflammation-induced pain sensitization in men and women: does sex matter in experimental endotoxemia?	**Study**	closeness	0.011084
Mindfulness-based stress reduction in relation to quality of life, mood, symptoms of stress, and immune parameters in breast and prostate cancer outpatients	**Study**	closeness	0.011084
Pain Catastrophizing and Quality of Life in Adults With Chronic Rhinosinusitis	**Study**	closeness	0.011084
Painful After-Sensations in Fibromyalgia are Linked to Catastrophizing and Differences in Brain Response in the Medial Temporal Lobe	**Study**	closeness	0.011084
Benefits of Yoga on IL-6: Findings from a Randomized Controlled Trial of Yoga for Depression	**Study**	closeness	0.010602
Intensive virtual reality and robotic based upper limb training compared to usual care, and associated cortical reorganization, in the acute and early sub-acute periods post-stroke: a feasibility study	**Study**	closeness	0.010602
Self-help Cognitive Behavioral Therapy Improves Health-Related Quality of Life for Inflammatory Bowel Disease Patients: A Randomized Controlled Effectiveness Trial	**Study**	closeness	0.010602
The effect of threat information on acquisition, extinction, and reinstatement of experimentally conditioned fear of movement-related pain	**Study**	closeness	0.010602
*PAIN RESPONSES*	**Topics**	closeness	0.183614
*Yoga Efficacy*	**Topics**	closeness	0.167711
*Pain and Inflammation*	**Topics**	closeness	0.141446
*Yoga and Pain Response*	**Topics**	closeness	0.109398
Rewriting Pain	**Topics**	closeness	0.076145
Yoga Cultural	**Topics**	closeness	0.064578
Complex Systems and Pain	**Topics**	closeness	0.014578

#### 4.2.3. Degree centrality and indegree

The measurement of degree centrality, or degree, involves a basic, undirected count of the total connections linked to a vertex. It is solely based in quantity; the quality of connections does not affect the value. Degree centrality can be useful for identifying popular connectors or local hubs, but it does not necessarily reflect the behavior of the broader network. For the purposes of this study, degree centrality generally tracks studies which the largest number of relevant keywords. Indegree is a submetric of degree centrality that exclusively measures a node's incoming connections. Nodes that have disproportionate incoming connections tend to be destinations for information or have an output outside of the network.

As seen in Table 4, KEYWORDS were the largest group in degree centrality and the only group for indegree metrics. For both groups demographic identifiers were the highest ranking, followed by nodes related to yoga, pain, and pain pathways. Pain responses and yoga's efficacy were the highest ranking TOPICS. Only one STUDY was in the top 30, related to neurological measurements of pain.

**Table 4:** Tracking the studies or keywords that showed the highest value in this metric. The measurement of degree centrality, or degree, involves a basic, undirected count of the total connections linked to a vertex.

Label	Type	Metric	Value
HUMANS	Keyword	degree	408
FEMALE	Keyword	degree	181
MALE	Keyword	degree	149
ADULT	Keyword	degree	143
YOGA	Keyword	degree	127
MIDDLE AGED	Keyword	degree	120
PAIN	Keyword	degree	98
AGED	**Keyword**	degree	65
*PAIN MEASUREMENT*	**Keyword**	degree	65
*QUALITY OF LIFE*	**Keyword**	degree	65
*CHRONIC PAIN*	**Keyword**	degree	58
*YOUNG ADULT*	**Keyword**	degree	54
*CATASTROPHIZATION*	**Keyword**	degree	46
*MEDITATION*	**Keyword**	degree	45
*RANDOMIZED CONTROLLED TRIALS AS TOPIC*	**Keyword**	degree	45
*TREATMENT OUTCOME*	**Keyword**	degree	43
*DEPRESSION*	**Keyword**	degree	42
*SURVEYS AND QUESTIONNAIRES*	**Keyword**	degree	41
*PAIN THRESHOLD*	**Keyword**	degree	39
*ADOLESCENT*	**Keyword**	degree	37
INFLAMMATION	**Keyword**	degree	37
*CROSS-SECTIONAL STUDIES*	**Keyword**	degree	35
*ANXIETY*	**Keyword**	degree	34
*CENTRAL NERVOUS SYSTEM SENSITIZATION*	**Keyword**	degree	33
*EXERCISE*	**Keyword**	degree	31
Angiotensin II Triggers Peripheral Macrophage-to-Sensory Neuron Redox Crosstalk to Elicit Pain	**Study**	degree	32
*PAIN RESPONSES*	**Topics**	degree	113
*Yoga Efficacy*	**Topics**	degree	110
*Pain and Inflammation*	**Topics**	degree	74
*Yoga and Pain Response*	**Topics**	degree	56
*HUMANS*	**Keyword**	indegree	408
*FEMALE*	**Keyword**	indegree	181
*MALE*	**Keyword**	indegree	149
*ADULT*	**Keyword**	indegree	143
*YOGA*	**Keyword**	indegree	127
*MIDDLE AGED*	**Keyword**	indegree	120
PAIN	**Keyword**	indegree	98
*AGED*	**Keyword**	indegree	65
*PAIN MEASUREMENT*	**Keyword**	indegree	65
*QUALITY OF LIFE*	**Keyword**	indegree	65
*CHRONIC PAIN*	**Keyword**	indegree	58
*YOUNG ADULT*	**Keyword**	indegree	54
*CATASTROPHIZATION*	**Keyword**	indegree	46
*MEDITATION*	**Keyword**	indegree	45
*RANDOMIZED CONTROLLED TRIALS AS TOPIC*	**Keyword**	indegree	45
*TREATMENT OUTCOME*	**Keyword**	indegree	43
*DEPRESSION*	**Keyword**	indegree	42
*SURVEYS AND QUESTIONNAIRES*	**Keyword**	indegree	41
*PAIN THRESHOLD*	**Keyword**	indegree	39
*ADOLESCENT*	**Keyword**	indegree	37
INFLAMMATION	**Keyword**	indegree	37
*CROSS-SECTIONAL STUDIES*	**Keyword**	indegree	35
*ANXIETY*	**Keyword**	indegree	34
*CENTRAL NERVOUS SYSTEM SENSITIZATION*	**Keyword**	indegree	33
COMPLEMENTARY THERAPIES	**Keyword**	indegree	31
*EXERCISE*	**Keyword**	indegree	31
ANIMALS	**Keyword**	indegree	30
MINDFULNESS	**Keyword**	indegree	24
EXERCISE THERAPY	**Keyword**	indegree	23
STRESS, PSYCHOLOGICAL	**Keyword**	indegree	23

#### 4.2.4. Eigenvector

Where degree centrality strictly measures quantity, and closeness centrality measures quantity with a small influence of quality, eigenvector centrality emphasizes quality of connection over quantity. Eigenvector values measure how well connected any given vertex is to the other most well-connected vertices. In general vertices with high eigenvector values reflect the leading edge of a network. Though they may not be as widely connected as other values, they tend to have disproportionate influence on the system. In the context of this study, eigenvector centrality is associated with nonlinear connections between areas of study.

As seen in Table 5, STUDIES had some of the highest value eigenvector nodes, and were nearly all related to biological complex systems. KEYWORDS were the largest block of high ranking eigenvector nodes, with all the top nodes being related to complex systems and modeling. Immune and inflammatory systems were next, followed by nodes related to pain, then several related to biology and physics. There was one TOPIC node, referencing complex systems.

**Table 5:** Tracking the studies or keywords that showed the highest value in this metric. Eigenvector values measure how well connected any given vertex is to the other most well-connected vertices.

Label	Type	Metric	Value
MODELING, COMPLEX SYSTEM	Keyword	eigenvector	0.030769
QUANTITATIVE BIOLOGY—QUANTITATIVE METHODS	Keyword	eigenvector	0.030769
BIOLOGICAL SYSTEM	**Keyword**	eigenvector	0.023077
MODELING, BIOLOGICAL	**Keyword**	eigenvector	0.023077
NONLINEAR SCIENCES—ADAPTATION AND SELF-ORGANIZING SYSTEMS	**Keyword**	eigenvector	0.023077
QUANTITATIVE BIOLOGY—NEURONS AND COGNITION	**Keyword**	eigenvector	0.023077
COMPLEX ADAPTIVE SYSTEM	**Keyword**	eigenvector	0.015385
NONLINEAR SCIENCES—CHAOTIC DYNAMICS	**Keyword**	eigenvector	0.015385
COMPUTER SCIENCE—SYMBOLIC COMPUTATION	**Keyword**	eigenvector	0.007692
IMMUNE SYSTEM	**Keyword**	eigenvector	0.007692
INFLAMMATION	**Keyword**	eigenvector	0.007692
MATHEMATICS—ALGEBRAIC GEOMETRY	**Keyword**	eigenvector	0.007692
MATHEMATICS—DYNAMICAL SYSTEMS	**Keyword**	eigenvector	0.007692
PAIN	**Keyword**	eigenvector	0.007692
PAIN RESPONSE	**Keyword**	eigenvector	0.007692
PHYSICS—BIOLOGICAL PHYSICS	**Keyword**	eigenvector	0.007692
PHYSICS—CHEMICAL PHYSICS	**Keyword**	eigenvector	0.007692
QUANTITATIVE BIOLOGY	**Keyword**	eigenvector	0.007692
QUANTITATIVE BIOLOGY—CELL BEHAVIOR	**Keyword**	eigenvector	0.007692
QUANTITATIVE BIOLOGY—TISSUES AND ORGANS	**Keyword**	eigenvector	0.007692
A Dynamical Similarity Approach to the Foundations of Complexity and Coordination in Multiscale Systems	**Study**	eigenvector	0.069231
Applying Complexity Theory to a Dynamical Process Model of the Development of Pathological Belief Systems	**Study**	eigenvector	0.069231
Complexity of Model Testing for Dynamical Systems with Toric Steady States	**Study**	eigenvector	0.069231
Designer dynamics through chaotic traps: Controlling complex behavior in driven nonlinear systems	**Study**	eigenvector	0.069231
Extreme value theory of evolving phenomena in complex dynamical systems: firing cascades in a model of neural network	**Study**	eigenvector	0.069231
Forecasting transitions in systems with high dimensional stochastic complex dynamics: A Linear Stability Analysis of the Tangled Nature Model	**Study**	eigenvector	0.069231
Life as Complex Systems—Viewpoint from Intra-Inter Dynamics	**Study**	eigenvector	0.069231
Small Open Chemical Systems Theory and Its Implications to Darwinian Evolutionary Dynamics, Complex Self-Organization and Beyond	**Study**	eigenvector	0.069231
Understanding and Modelling the Complexity of the Immune System: Systems Biology for Integration and Dynamical Reconstruction of Lymphocyte Multi-Scale Dynamics	**Study**	eigenvector	0.069231
*Complex System*	**Topics**	eigenvector	0.069231

#### 4.2.5. Reach efficiency

Reach measures the portion of the network within two steps of an element. In general, elements with high reach can spread information through the network through close friend-of-a-friend contacts. Reach efficiency normalizes reach by dividing it by size (number of neighbors). In general, elements with high reach efficiency are less connected but gain more exposure through each direct relationship. Reach efficiency is useful for determining influence as well as indicating how coherent and consistent that influence is.

As seen in Table 6, reach efficiency was mostly split between KEYWORDS and STUDIES in terms of quantity, but the highest values were among STUDIES. The leading STUDY nodes all involved complex systems and pain. The leading KEYWORDS were highly heterogeneous and patterns were not readily identifiable.

**Table 6:** Tracking the studies or keywords that showed the highest value in this metric. Reach efficiency normalizes reach by dividing it by size (number of neighbors).

Label	Type	Metric	Value
ACCIDENTS, HOME	Keyword	reach-efficiency	0.000241
ACQUIRED BRAIN INJURY	Keyword	reach-efficiency	0.000241
ANALYSIS OF VARIANCE	**Keyword**	reach-efficiency	0.000241
ANTERIOR CINGULATE CORTEX	**Keyword**	reach-efficiency	0.000241
CANCER RELATED PAIN	**Keyword**	reach-efficiency	0.000241
CELLS, CULTURED	**Keyword**	reach-efficiency	0.000241
COUNSELING	**Keyword**	reach-efficiency	0.000241
GASTROESOPHAGEAL REFLUX	**Keyword**	reach-efficiency	0.000241
LASERS	**Keyword**	reach-efficiency	0.000241
LEADERSHIP	**Keyword**	reach-efficiency	0.000241
PSYCHOLOGICAL TREATMENT	**Keyword**	reach-efficiency	0.000241
REPETITIVE SENSORY STIMULATION	**Keyword**	reach-efficiency	0.000241
SUPINE POSITION	**Keyword**	reach-efficiency	0.000241
A Dynamical Similarity Approach to the Foundations of Complexity and Coordination in Multiscale Systems	**Study**	reach-efficiency	0.001252
COMPLEX SYSTEMS	**Study**	reach-efficiency	0.001252
Forecasting transitions in systems with high dimensional stochastic complex dynamics: A Linear Stability Analysis of the Tangled Nature Model	**Study**	reach-efficiency	0.001252
Life as Complex Systems—Viewpoint from Intra-Inter Dynamics	**Study**	reach-efficiency	0.001252
Applying Complexity Theory to a Dynamical Process Model of the Development of Pathological Belief Systems	**Study**	reach-efficiency	0.001124
Designer dynamics through chaotic traps: Controlling complex behavior in driven nonlinear systems	**Study**	reach-efficiency	0.001124
Complexity of Model Testing for Dynamical Systems with Toric Steady States	**Study**	reach-efficiency	0.001032
CRPS: A contingent hypothesis with prostaglandins as crucial conversion factor	**Study**	reach-efficiency	0.001032
Small Open Chemical Systems Theory and Its Implications to Darwinian Evolutionary Dynamics, Complex Self-Organization and Beyond	**Study**	reach-efficiency	0.001032
Extreme value theory of evolving phenomena in complex dynamical systems: firing cascades in a model of neural network	**Study**	reach-efficiency	0.000963
Structure and dynamics of dynorphin peptide and its receptor	**Study**	reach-efficiency	0.000963
Painful intelligence: What AI can tell us about human suffering	**Study**	reach-efficiency	0.00091
Understanding and Modelling the Complexity of the Immune System: Systems Biology for Integration and Dynamical Reconstruction of Lymphocyte Multi-Scale Dynamics	**Study**	reach-efficiency	0.00091
Hormesis, adaptation, and the sandpile model	**Study**	reach-efficiency	0.000867
Pain pathogenesis in rheumatoid arthritis—what have we learned from animal models	**Study**	reach-efficiency	0.000867
A Deep Learning Approach to Diagnosing Multiple Sclerosis from Smartphone Data	**Study**	reach-efficiency	0.000803
Complex adaptive systems allostasis in fibromyalgia	**Study**	reach-efficiency	0.000803

#### 4.2.6. Synthesis

All nodes (studies, topics, and keywords) were mapped through the network analysis and the highest 30 values for each network metric isolated. Every node was cross-referenced and any node that had multiple high network metric values were highlighted for examination. Leaders in this synthesis will be helpful in identifying the similarities in keywords and patterns of topics between fields that are not usually linked.

As seen in Table 7, several nodes were high ranking across three metrics, potentially acting as indicators of the broader pattern of the research.

**Table 7:** Nodes that were high-valued in 3 or more different metrics.

Label	Type
A Dynamical Similarity Approach to the Foundations of Complexity and Coordination in Multiscale Systems	Study
Angiotensin II Triggers Peripheral Macrophage-to-Sensory Neuron Redox Crosstalk to Elicit Pain	Study
Applying Complexity Theory to a Dynamical Process Model of the Development of Pathological Belief Systems	Study
Complexity of Model Testing for Dynamical Systems with Toric Steady States	Study
Designer dynamics through chaotic traps: Controlling complex behavior in driven nonlinear systems	Study
Extreme value theory of evolving phenomena in complex dynamical systems: firing cascades in a model of neural network	Study
Forecasting transitions in systems with high dimensional stochastic complex dynamics: A Linear Stability Analysis of the Tangled Nature Model	Study
INFLAMMATION	Keyword
Life as Complex Systems—Viewpoint from Intra-Inter Dynamics	Study
PAIN	Keyword
Small Open Chemical Systems Theory and Its Implications to Darwinian Evolutionary Dynamics, Complex Self-Organization and Beyond	Study
Understanding and Modelling the Complexity of the Immune System: Systems Biology for Integration and Dynamical Reconstruction of Lymphocyte Multi-Scale Dynamics	Study

The majority of the most influential nodes were STUDIES relating to complex systems and nodes relating to pain pathways. This suggests there are significant intersections between the study of complex systems and pain.

As seen in Table 8, a larger number of nodes were leaders among two metrics, and could be considered part of a larger, defining pattern between the fields of pain management and mind-body therapies.

**Table 8:** Nodes that were high-valued in 2 different metrics.

Label	Type
A Mechanism-Based Approach to the Management of Osteoarthritis Pain	Study
ADOLESCENT	Keyword
ADULT	**Keyword**
*AGED*	**Keyword**
*ANXIETY*	**Keyword**
*Assessing for unique immunomodulatory and neuroplastic profiles of physical activity subtypes: a focus on psychiatric disorders*	**Study**
*CATASTROPHIZATION*	**Keyword**
*CENTRAL NERVOUS SYSTEM SENSITIZATION*	**Keyword**
*CHRONIC PAIN*	**Keyword**
*Common Brain Mechanisms of Chronic Pain and Addiction*	**Study**
*Complex System*	**Topics**
*CROSS-SECTIONAL STUDIES*	**Keyword**
*DEPRESSION*	**Keyword**
*EXERCISE*	**Keyword**
*FEMALE*	**Keyword**
*HUMANS*	**Keyword**
*Immediate preoperative outcomes of pain neuroscience education for patients undergoing total knee arthroplasty: A case series*	**Study**
*Influence of a periodized circuit training protocol on intermuscular adipose tissue of patients with knee osteoarthritis: protocol for a randomized controlled trial*	**Study**
*Low- Versus High-Intensity Plyometric Exercise During Rehabilitation After Anterior Cruciate Ligament Reconstruction*	**Study**
*MALE*	**Keyword**
*MEDITATION*	**Keyword**
*MIDDLE AGED*	**Keyword**
*Pain and Inflammation*	**Topics**
*PAIN MEASUREMENT*	**Keyword**
*PAIN RESPONSES*	**Topics**
*PAIN THRESHOLD*	**Keyword**
*Psychological processing in chronic pain: a neural systems approach*	**Study**
*QUALITY OF LIFE*	**Keyword**
*RANDOMIZED CONTROLLED TRIALS AS TOPIC*	**Keyword**
*SURVEYS AND QUESTIONNAIRES*	**Keyword**
*TREATMENT OUTCOME*	**Keyword**
*YOGA*	**Keyword**
*Yoga and Pain Response*	**Topics**
*Yoga Efficacy*	**Topics**
*YOUNG ADULT*	**Keyword**

Setting aside demographic identifier KEYWORDS, the nodes that were leading in two separate metrics tend to involve pain management, chronic pain, neuroscience, pain education, sensitization/catastrophization, and yoga or meditation. This suggests that the broader literature review highlights the close connections between chronic pain and mind-body therapies.

Combining the blocks of synthesized findings provides evidence that the fields of study regarding complex systems, pain management, and mind-body therapies share many of the same topics, keywords, and published studies. The literature review suggests the fields share significant patterns.

### 4.3. Functional systems mapping results

**Figure 4:**
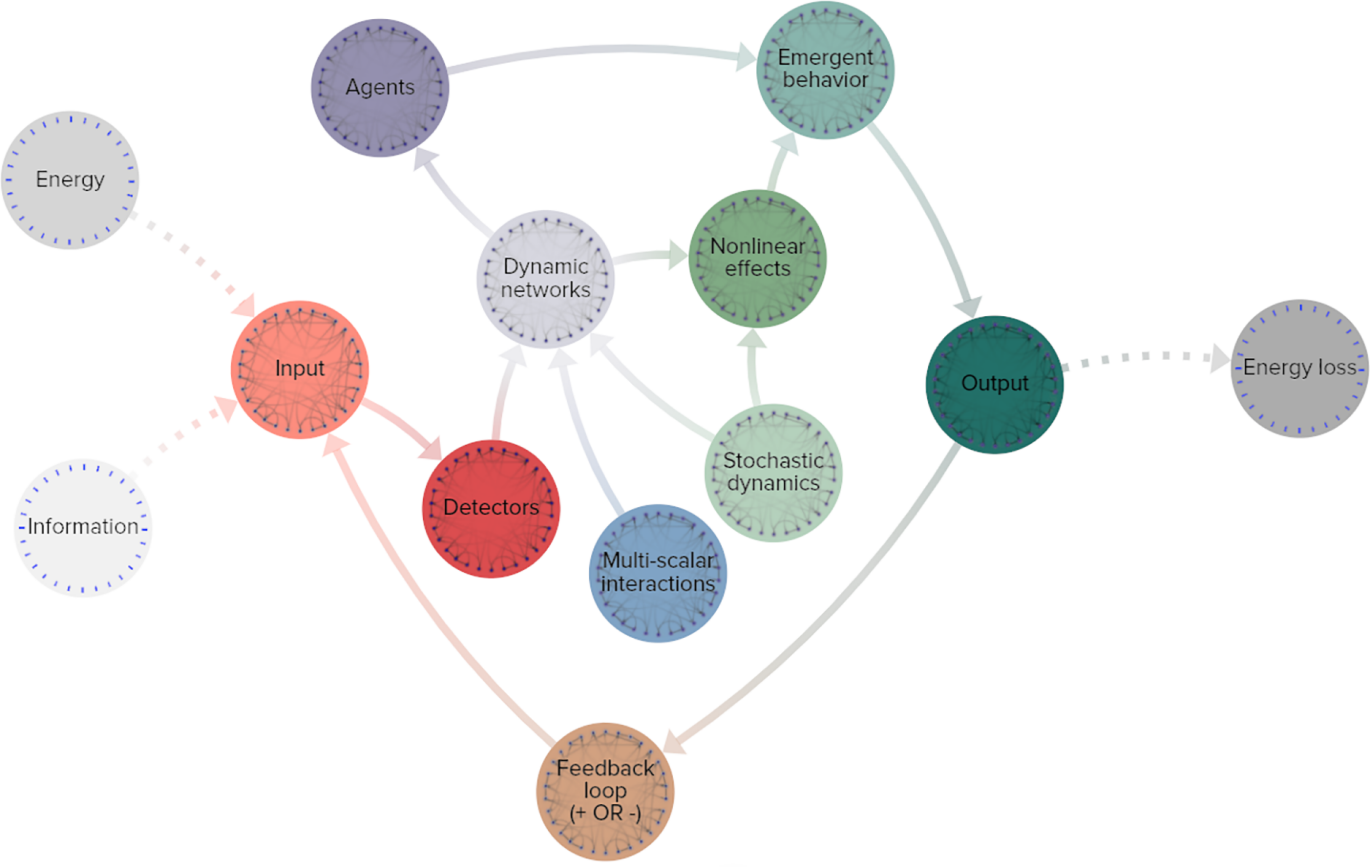
Simplified functional map of a complex adaptive system. Grey-scale nodes: outside the system, orange: input stimuli, red: functional detection, blue: multi-scale networks, light green: unpredictable effects, violet: dynamic networks, purple: active agents of change, dark teal: effects of system change, brown: output leading to input in a feedback loop.

Figure 4 reflects a basic map of the essential functions and components of a complex adaptive system. Any potential CAS should possess elements that fulfill every function and operate at a net energy loss.

1. **Energy and information**: the external environment interacting with the system
2. **Input**: a means of absorbing this energy or information transfers it into the interconnected system
3. **Detectors**: identify and react to the new input, changing the behavior of the system
4. **Dynamic networks**: changing relationships in response to the detector’s signals throughout the system, these networks include
  a. **Multi scalar interactions**: simple mechanics on many different scales
  b. **Stochastic dynamics**: unpredictable, highly sensitive reactions and feedback loops
5. **Agents**: mechanisms by which the system actively modulates and adjusts behavior in response to the changes in the dynamic network
6. **Nonlinear effects**: a secondary feature of the dynamic network interactions includes nonlinear effects, systemic and often disproportionate reactions to changes
7. **Emergent behavior**: the combination of active adjustments by agents and unpredictable nonlinear effects results in emergent behavior that is inextricably part of a holistic pattern and cannot be reliably modeled in isolation
8. **Output**: the behavioral changes of the system often interact with environment outside the system and modify its relationship with it
  a. **Feedback loops**: the changes in output can shift the way the initial input is received, altering the entire set of reactions in either positive or negative feedback loops
  b. **Energy loss**: the agency and behavior of the system expends energy, usually back into the outside environment, in what is considered an open system

#### 4.3.1. Pain response map

The array of pain response pathways comprise every function of a complex adaptive system and the overall mechanisms are far from energetic equilibrium.

**Figure 5:**
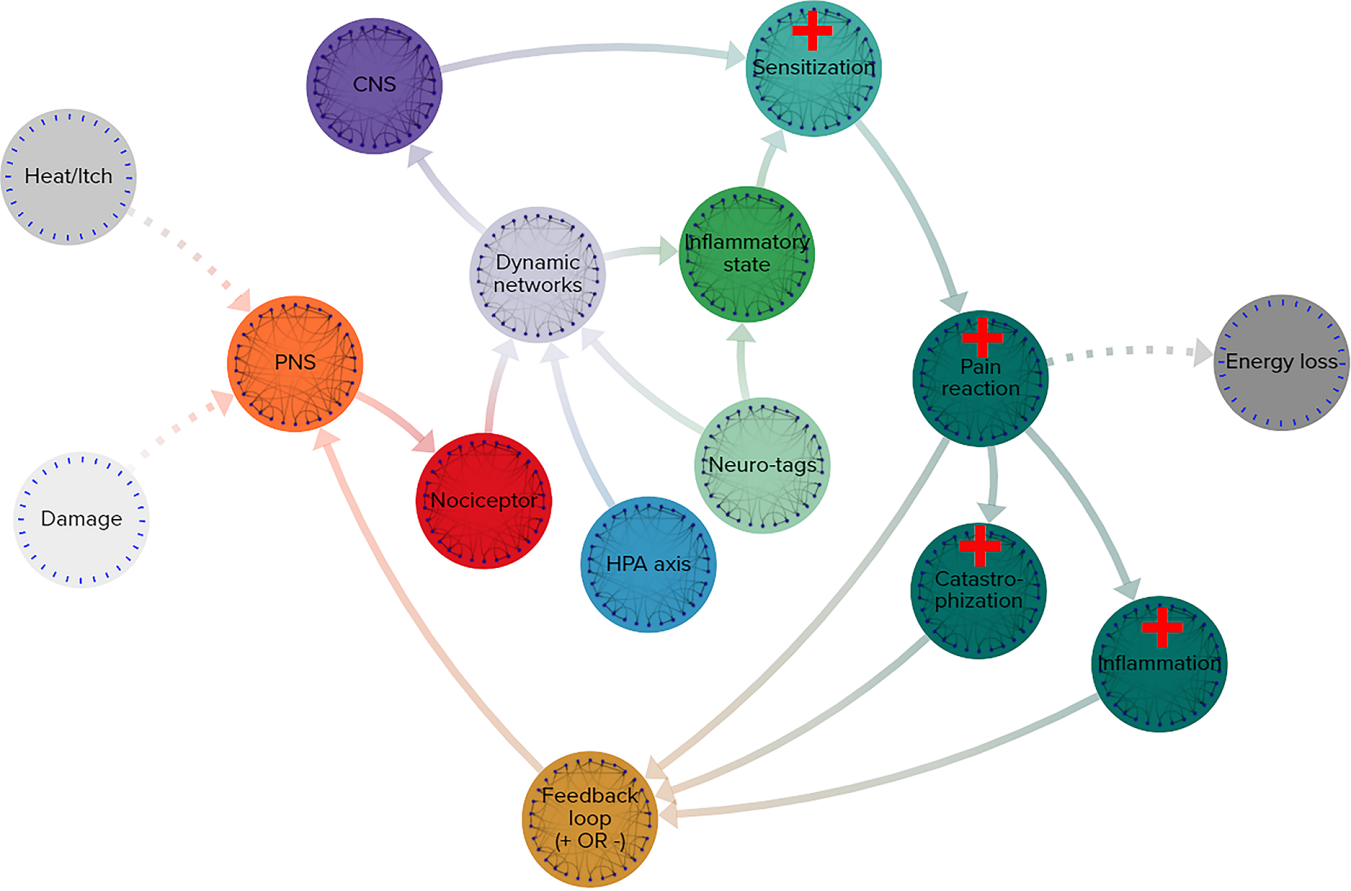
Simplified functional map of pain response system. Grey-scale nodes: outside the system, orange: input stimuli, red: functional detection, blue: multi-scale networks, light green: unpredictable effects, violet: dynamic networks, purple: active agents of change, dark teal: effects of system change, brown: output leading to input in a feedback loop.

1. **Energy and information**: events related to heat, itch, or damage affect the system from the outside environment
2. **Input**: the peripheral nervous system is involved with receiving the initial input signals (Figure 5)
3. **Detectors**: nociceptors identify the sensations and sends signals toward the central nervous system
4. **Dynamic networks**: cascading effects across nociceptive, sensory, and other networks in reaction to the pain responses throughout the body, these networks include
  a. **Multi scalar interactions**: an example of a multi-scale network influenced by pain signals is the hypothalamic-pituitary-adrenal axis (HPA axis), which regulates neuroendocrine responses that range from digestive networks to immune responses
  b. **Stochastic dynamics**: one of the many examples of unpredictable reactions can be neuro-tagging, when interoceptive and exteroceptive data combine with nociceptive information to classify sensory signals to the rest of the body
5. **Agents**: the central nervous system is a clear vehicle serving as an agent in pain response systems
6. **Nonlinear effects**: the inflammatory load and reaction of the body has a highly significant influence on pain outcomes, even small differences in inflammatory states can cause chain reactions with enormous implications
7. **Emergent behavior**: sensitization to pain signaling is influenced by a large number of complex variables and in turn produces numerous outputs in the body’s pain management and healing response that cannot be precisely modeled without taking the entire pain response system into account
8. **Output**: the body’s various pain reactions are both internal and external-facing
  a. **Feedback loops**: combinations of inflammatory reactions and catastrophization can increase sensitization to input and thereby increase pain reaction, inflammatory responses, and catastrophization in systemic feedback loops
  b. **Energy loss**: the functioning of the pain response system relies on cellular energy and caloric expenditure, both of which are indicative of an energetically open system

#### 4.3.2. Yoga therapy map

The components of yogic practice comprise every function of a complex adaptive system and the overall mechanisms are far from energetic equilibrium.

The array of pain response pathways comprise every function of a complex adaptive system and the overall mechanisms are far from energetic equilibrium.

**Figure 6:**
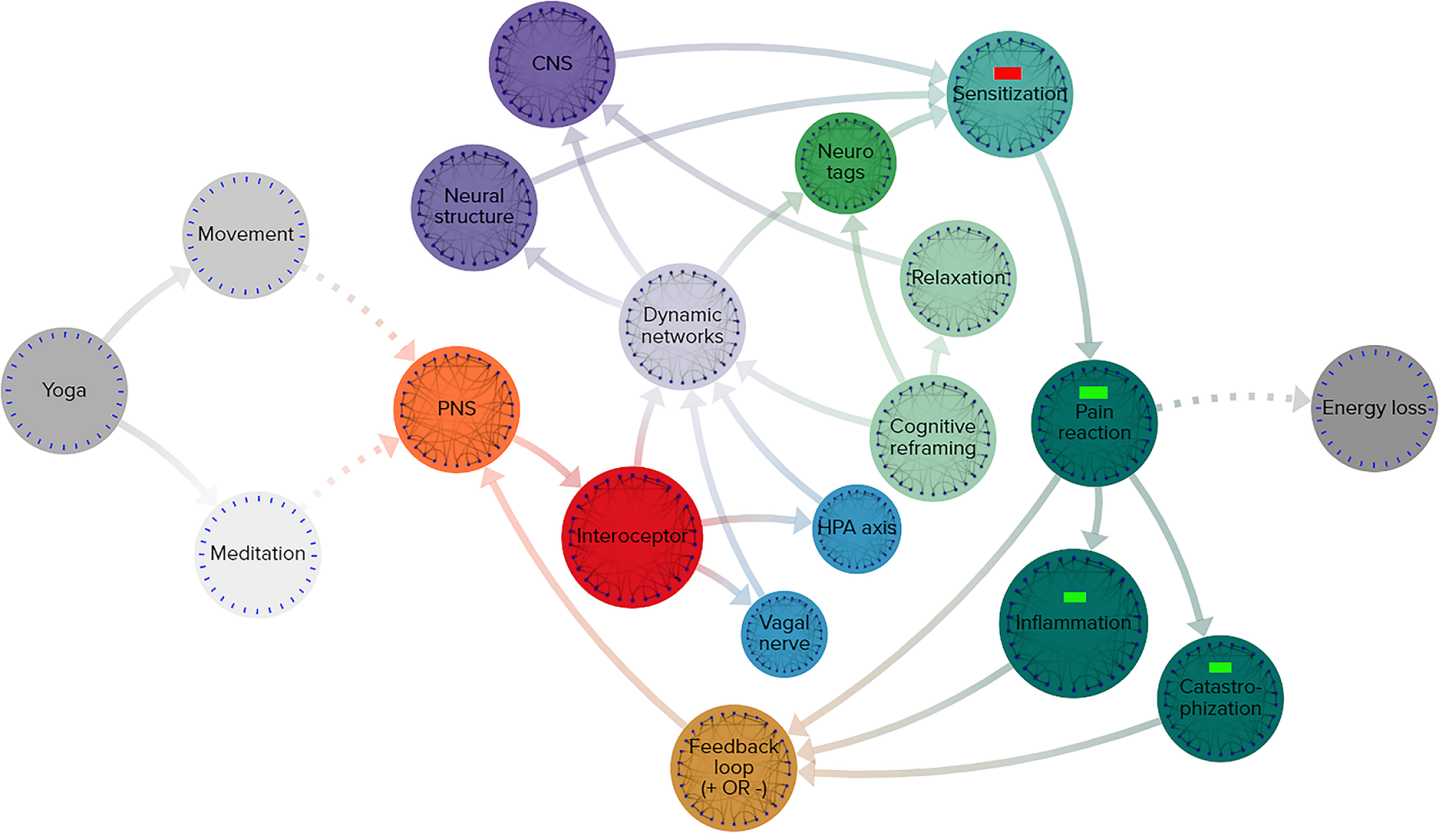
Simplified functional map of neurobiological effects of yoga practice. Grey-scale nodes: outside the system, orange: input stimuli, red: functional detection, blue: multi-scale networks, light green: unpredictable effects, violet: dynamic networks, purple: active agents of change, dark teal: effects of system change, brown: output leading to input in a feedback loop.

1. **Energy and information**: the practice of yoga involves both physical movement and mental activities, comprising both energy and information (Figure 6)
2. **Input**: the peripheral nervous system is involved with receiving the initial input signals
3. **Detectors**: interoceptors identify the internal sensations and sends signals toward the central nervous system
4. **Dynamic networks**: cascading effects across interoceptive, nociceptive, sensory, and other networks in reaction to the pain responses throughout the body, these networks include
  a. **Multi scalar interactions**: an example of a multi-scale network influenced by yoga is the hypothalamic-pituitary-adrenal axis (HPA axis), which regulates neuroendocrine responses that range from digestive networks to immune responses, and the tone of the vagal nerve, both of which impact networks on multiple scales
  b. **Stochastic dynamics**: one of the many examples of unpredictable reactions can occur during cognitive reframing, when new interoceptive and exteroceptive data combine with existing nociceptive information to reclassify sensory signals to the rest of the body
5. **Agents**: the central nervous system is a clear vehicle serving as an agent in reactions to the physical and mental activities of yoga, while alterations to the brain’s neural structure through repeated practice serve as agents of change
6. **Nonlinear effects**: any alterations in neuro-tagging have a highly significant influence on pain management, even small differences in sensitization signals can cause chain reactions with enormous implications
7. **Emergent behavior**: sensitization to pain signaling is influenced by a large number of complex variables and in turn produces numerous outputs in the body’s pain management and healing response, meaning yoga’s full impact on pain cannot be precisely modeled without taking the entire mind-body therapeutic system into account
8. **Output**: the body’s various reactions to yoga practice are both internal and external-facing
  a. **Feedback loops**: combinations of reductions in inflammatory and catastrophization can decrease sensitization to input and thereby pain reactions, inflammatory responses, and catastrophization in systemic feedback loops
  b. **Energy loss**: the practice of yoga relies on cellular energy and caloric expenditure, both of which are indicative of an energetically open system

## 5. Analysis

The examination of the complexity of yoga’s role in pain management was conducted in three parts.

• Evidence overview: a textual discussion of the published research relating yoga and pain responses within the framework of a complex system
• Network analysis: determining the strength of connection between different topics and research articles related to yoga, pain, and complex systems
• Functional systems mapping: cross-referencing the core components of a complex system with the behavior of pain responses and yoga as pain management

### 5.1. Evidence overviews

The efficacy and operational pathways of yoga are sufficiently documented to allow comparisons to the established pathways of the pain response system. A review of existing literature suggests that both pain stimuli and the practice of yoga interact with many of the same systems in the peripheral and central nervous system, with the therapeutic effects of yoga often addressing the most detrimental side-effects of pain responses.

From a systems perspective the evidence overview supports the case that pain responses and yoga practices are each complex adaptive systems. Each system involves a range of physiological and neurological interactions that lead to reactions exhibiting all the defining characteristics of a complex adaptive system. Pain responses have been well established as a complex adaptive system, the evidence overview confirms this. Yoga's definition as a CAS is novel but as well evidenced as pain responses’. Due to the multimodal nature of yoga, this definition appears to apply both in connection with pain management and in isolation.

From a functional perspective this information indicates yoga to be a complex system that is an effective method of treating pain. Given the complex manifestations of pain in neurological, psychological, and inflammatory contexts, a systemic intervention could be of particular value.

### 5.2. Network analyses

The network analyses of the selected body of studies and their associated keywords sought to identify patterns in the findings relating to pain, yoga, and complex systems. The frequency of keyword use in studies across various topics will be used to extrapolate the predominance of different topics in the research. Findings from the network analysis were compiled into a cluster graph and the relevant metrics emphasized.

**Figure 7:**
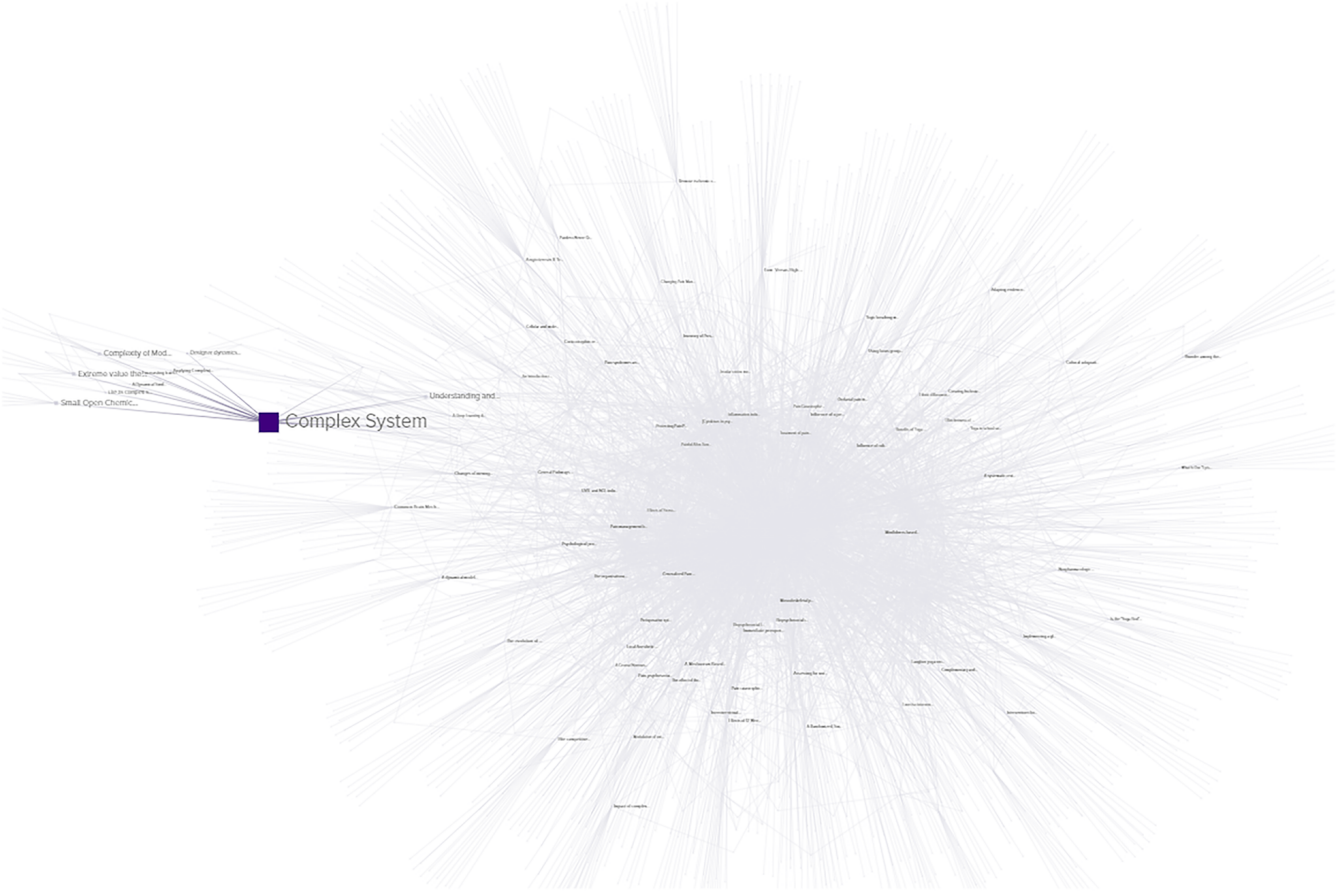
Betweenness network analysis visualization, demonstrating nodes and connections between TOPICS, STUDIES, and KEYWORDS. Nodes with higher values are larger and darker in color.

**Figure 8:**
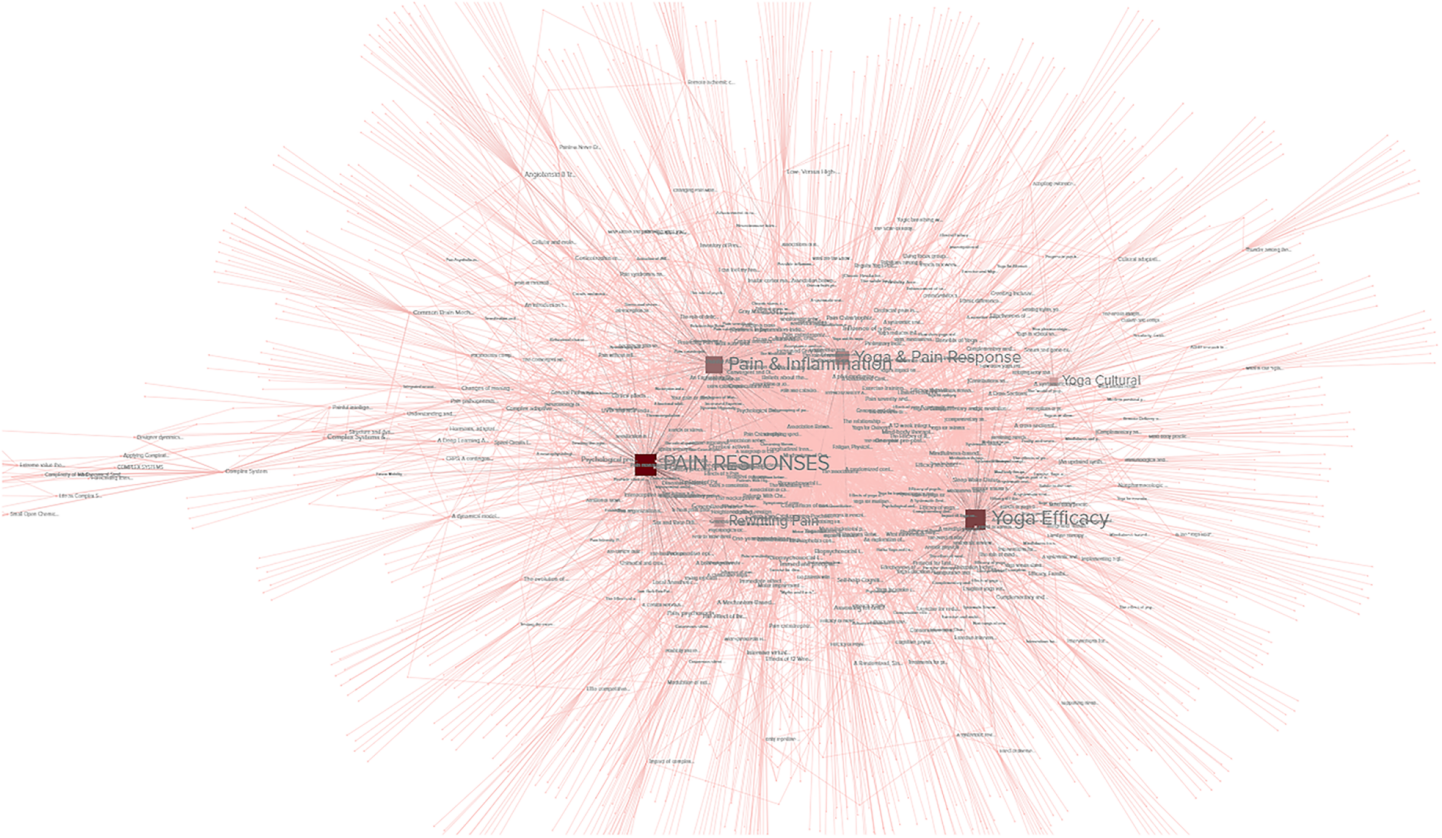
Closeness network analysis visualization, demonstrating nodes and connections between TOPICS, STUDIES, and KEYWORDS. Nodes with higher values are larger and darker in color.

**Figure 9:**
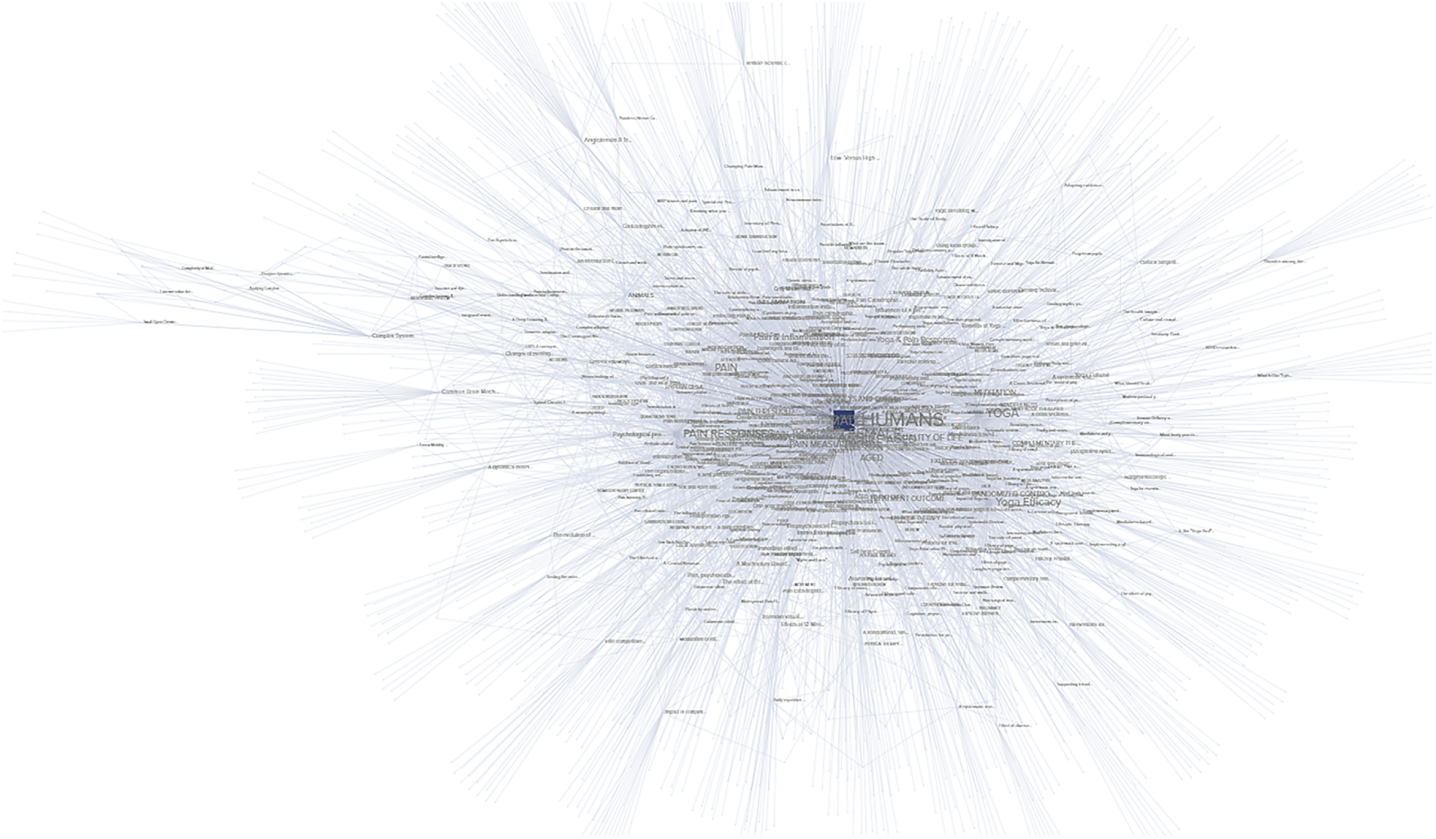
Degree network analysis visualization, demonstrating nodes and connections between TOPICS, STUDIES, and KEYWORDS. Nodes with higher values are larger and darker in color.

**Figure 10:**
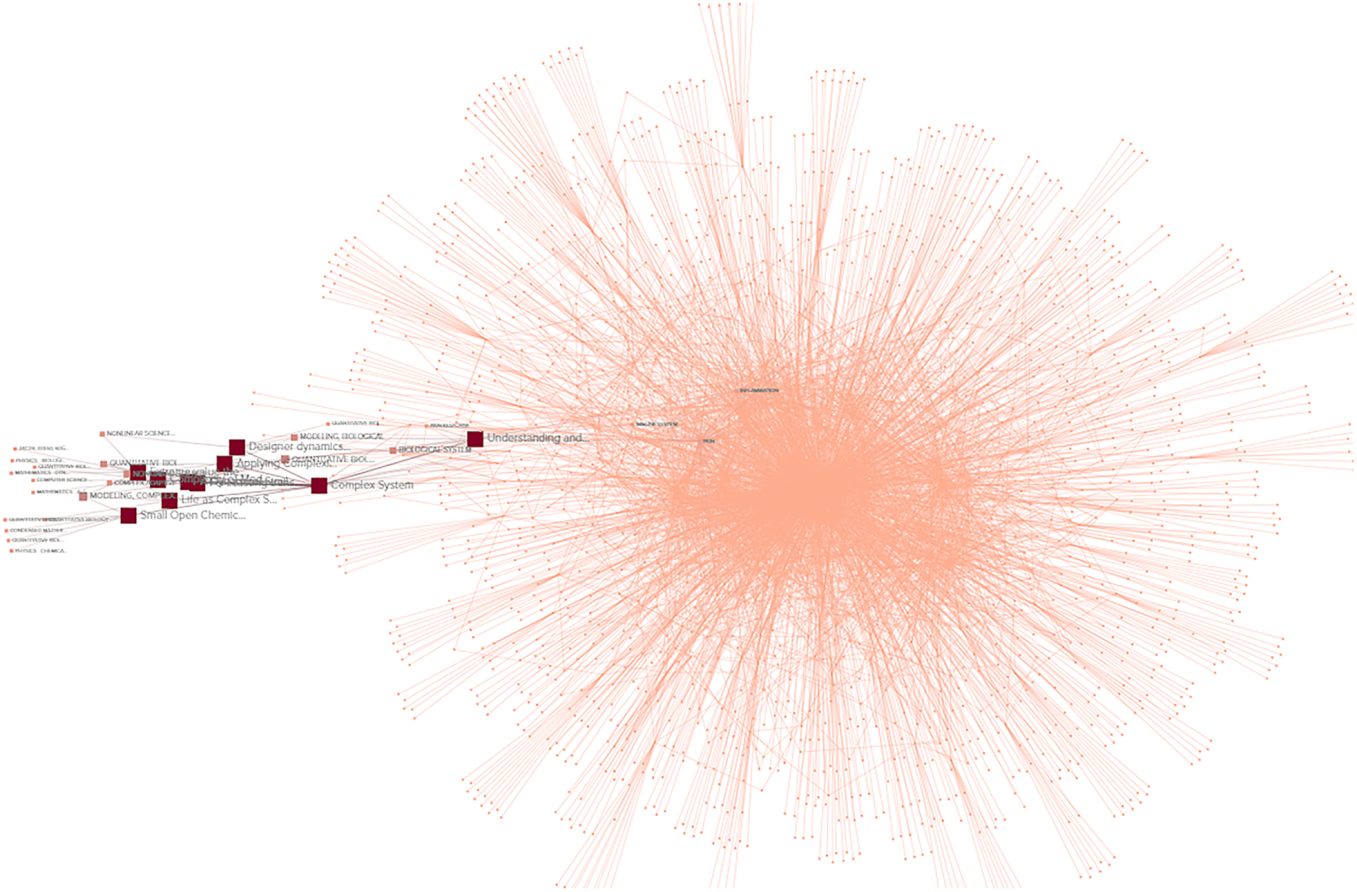
Eigenvector network analysis visualization, demonstrating nodes and connections between TOPICS, STUDIES, and KEYWORDS. Nodes with higher values are larger and darker in color.

**Figure 11:**
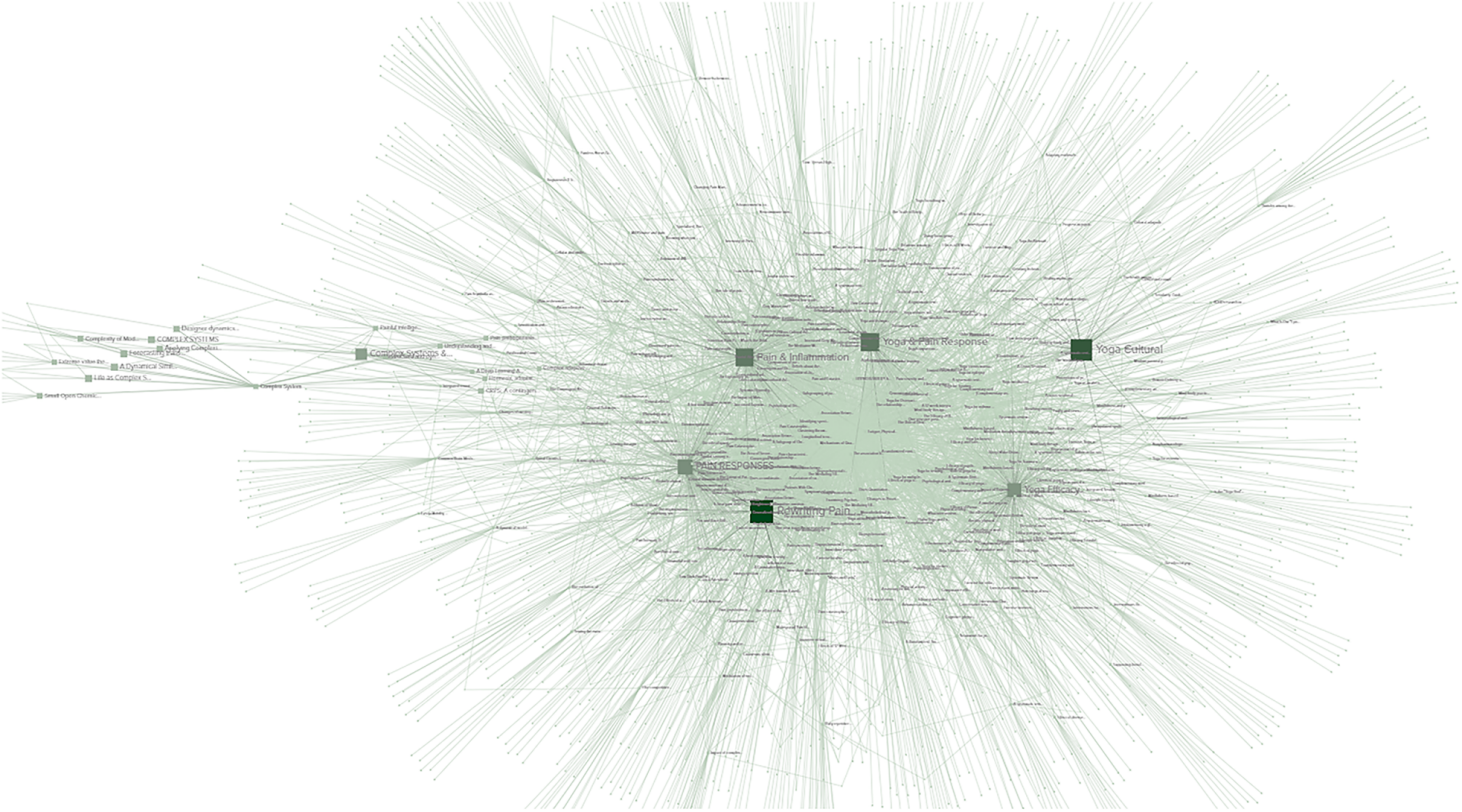
Reach efficiency network analysis visualization, demonstrating nodes and connections between TOPICS, STUDIES, and KEYWORDS. Nodes with higher values are larger and darker in color.

• **Betweenness**: as seen in Figure 7, given the relatively isolated nature of the keywords related to complex systems, it is unsurprising that a metric tracking traffic would exhibit high values related to complex systems, much like a bottleneck increases pressure. The themes covered in the highest betweenness values include (due to nodes that are irrelevant or apply to two or more themes, percentages may not total 100%):
  • Complex systems 33.3%: This suggests that while complex systems are not central to most studies relating to pain, the existing connections between the topics are heavily trafficked
  • Pain responses 30%: This topic was central to a significant number of connections, reflecting the importance of pain to many topics
  • Pain management 30%: This topic was central to a significant number of connections, reflecting the importance of pain management to many topics
  • Yoga 13.3%: Yoga was not as significant in betweenness, possibly due to the wide-ranging, less centralized nature of the studies.
• **Closeness**: as seen in Figure 8, this metric often identifies the dominant tendencies within a network and can be used to determine the most interdependent factors of the literature review. Predictably the highest values are clustered near the center of the graph, close to the highest concentration of connections. The themes covered in the highest closeness values include (due to nodes that are irrelevant or apply to two or more themes, percentages may not total 100%):
  • Complex systems 3.3%: Low values indicate that the concept of complex systems is not frequently incorporated into the most common studies
  • Pain responses 63.3%: As this is a widely studied and well-established field, it is expected that pain responses would dominate the trends of most common keywords
  • Pain management 30%: This topic was central to significant numbers of connections, for many of the same reasons as pain responses
  • Yoga 23.3%: Yoga exhibited significant values in closeness, possibly for the same reasons it scored low in betweenness: the broad, decentralized nature of many yoga studies touch on large numbers of trending topics
• **Degree centrality**: as seen in Figure 9, degree centrality is a simple quantitative metric and is prone to overvaluing diagnostic data. Many of the leading values in degree involved irrelevant terms and keywords, but the fact pain responses and management still ranked highly reinforces their importance to this network. The themes covered in the highest degree values include (due to nodes that are irrelevant or apply to two or more themes, percentages may not total 100%):
  • Complex systems 0%: No values indicate that the concept of complex systems is not directly connected to most studies
  • Pain responses 40%: As this is a widely studied and well-established field, it is expected that pain responses would be frequently connected to the most common keywords
  • Pain management 20%: This topic was central to significant numbers of connections, for many of the same reasons as pain responses
  • Yoga 16.6%: Yoga exhibited small values in degree centrality, suggesting that while certain aspects of yoga are highly connected, these aspects are often disparate and separated among studies
• **Eigenvector**: as seen in Figure 10, measurements of eigenvector values are particularly useful for identifying systems. This metric reveals the parts of a network that have the greatest nonlinear representation, suggesting topics that may not be the dominant trends but underpin and amplify them.
  • Complex systems 83.3%: The extremely high eigenvector value for topics related to complex systems reinforces its utility as a predictor of systems influence. Much like complex systems themselves, studies about complex systems are often disproportionately influential and relevant to multidisciplinary fields.
  • Pain responses 16.6%: The majority of the topics related to pain were also related to systems like the immune system or inflammatory response system
  • Pain management 0%: The lack of any pain management in the top eigenvector values was unexpected, given that it is a response to pain itself, which was represented
  • Yoga 0%: The lack of any yoga topics in the top eigenvector values was unexpected, given that it is a response to pain itself and a multidisciplinary approach in itself
• **Reach efficiency**: as seen in Figure 11, combining the qualitative analysis of eigenvector metrics with the simple quantitative metrics of degree centrality results in reach efficiency, which can provide mitigate some of the outliers in either approach. This is reflected in the more well-rounded findings of this study’s network analysis.
  • Complex systems 56.6%: The high reach efficiency value for topics related to complex systems suggests strong connections within just two degrees, as well as across the network
  • Pain responses 40%: The majority of the topics related to pain were also related to complex systems, suggesting systems approaches are increasingly relevant to the field
  • Pain management 10%: As a subset of pain research, it is logical that pain management would be represented to a significant but smaller degree than pain responses as a whole
  • Yoga 0%: The lack of any yoga topics in the top reach efficiency values was unexpected, given its close relationship to pain research
• **Synthesis**: Selecting the nodes that consistently ranked in the top metrics for two or three different metrics could provide insights to important topics and themes that are not obvious from a single measurement.
  • 3 metrics: Out of 12 nodes that were high ranking in at least 3 separate metrics, 75% were related to complex systems and 25% were related to pain responses, suggesting that at the highest levels complex systems are closely intertwined with pain research
  • 2 metrics: Out of 35 nodes that were high ranking in 2 separate metrics 20% were related to pain responses, 20% related to nodes similar to yoga, 17.1% related to pain management, and 3% related to complex systems. A notable observation is that nodes related to yoga and pain responses are equally represented, despite pain response nodes being far more common across each individual metric.

Network analysis showed pervasive patterns connecting pain responses, yoga, and complex system research. The most prevalent keywords in all three fields of study have numerous, strong associations.

### 5.3. Functional systems maps

The functional mapping of pain responses and yoga practice was conducted to identify whether there were functions possessed by either system analogous to the minimum requirements to be considered a complex adaptive system. Any potential CAS should possess elements that fulfill every one of these functions and operates at a net energy loss.

1) **Energy and information**
2) **Input**
3) **Detectors**
4) **Dynamic networks**
   (a) **Multi scalar interactions**
   (b) **Stochastic dynamics**
5) **Agents**
6) **Nonlinear effects**
7) **Emergent behavior**
8) **Output**
  (a) **Feedback loops**
  (b) **Energy loss**

Both the practice of yoga and the pain response system met every major definition of a complex adaptive system.

## 6. Conclusion

Three distinct investigative methods were used to examine the potential of yoga as a complex system of pain management. All three demonstrated evidence that the practice of yoga for pain is effective and behaves like a complex system.

• Evidence overview: yoga and pain operate along many of the same sensory pathways, making yoga a systemically effective form of pain management. Both pain responses and yoga practice demonstrate complex system behaviors.
• Network analysis: among 7 different metrics tracking research into pain, yoga, and complex systems, 3 metrics showed connections between all three, 3 showed connections between complex systems and pain, and 1 showed connections between yoga and pain. No metric demonstrated isolated values or a lack of connection. This suggests an interconnected, complex system.
• Functional system mapping: overlaying the mechanisms of pain responses and yoga onto a map of the core mechanisms required for a complex system revealed a consistent overlap. Yoga as pain management fulfills every requisite function to be considered a complex system, and regulates many of the same mechanisms affected by pain responses.

### 6.1. Limitations

The study of complex systems is necessarily nuanced and multifaceted. There are numerous aspects of complex systems that were not examined due to either a lack of data or expertise. This includes determining the presence and significance of strange attractors, functional systems models or modules, and detailed statistical analysis. Further examination of this topic by specialists in the field of complex systems is warranted and necessary to verify this study's broad implications.

Further, the practice of yoga is varied and cannot be generalized. Any metrics tracking the effects of yoga are informed by the particular tradition being practiced, the adherence of the practitioner, and the amount of time spent practicing. These factors make interpolation of the discrete features of yoga problematic.

### 6.2. Discussion

Evidence overviews established the practice of yoga as a viable pain management therapy that shared many of the characteristics of a complex adaptive system. Network analysis of 433 studies and 1,639 keywords identified pain responses and yoga-related topics as comparable across numerous metrics, suggesting a strong relationship and interconnected system. The greatest concentration of highly influential keywords indicate complex systems are the dominant, if indirect, connecting feature across studies, providing further evidence that pain response systems and yoga practice are both complex systems. Mapping the essential functions of complex adaptive systems onto pain responses and yoga practice demonstrated that both systems met every requirement of operational complex adaptive systems. It is notable that the functional mapping of yoga demonstrated interactions with nearly every one of the body's systems that pain impacted.

Recent reviews have supported the role of yoga as a pain management intervention, but since most research has focused on isolated, usually physical components of yoga rather than systemic mind-body effects, multiple forms of analysis were considered necessary to examine the novel hypothesis of this study (68). These diverse methods all support considering yoga a complex adaptive system that exhibits unique interactions with the pain response system. Much like the consequences of pain can have pervasive, unpredictable effects on homeostasis, it should be considered that the practice of yoga could likewise have systemic, indirect impacts. This is especially relevant when considering chronic pain, long-term interventions, and quality of life.

### 6.3. Implications

Designation as a complex adaptive system entails significant changes in how the effects of an intervention are tracked and interpreted. Complex adaptive systems are emergent phenomenon that cannot be reduced to simple, linear interactions.

In regards to research, an understanding of this dynamic could significantly improve study of mind-body therapies like yoga, shifting the attention from the presumed mechanism in isolation to the emergent effect on the total health of the patient. This nonlinear perspective may address the often-cited unpredictability in yoga research and shift methodologies from short-term metrics to measuring long-term systemic changes. The wide-ranging benefits of yoga for pain management and similarity in function to broader behavioral health interventions suggest a similar approach to other mind-body therapies is warranted.

At the level of direct patient interventions, this study provides an overview of the evidence indicating yoga is a viable option for pain management. Further, yoga may be uniquely suited to treat systemic chronic issues as a result of operating as a holistic rather than discrete intervention. Another intervention-centric benefit to this may lie in reorienting recommendations by health professionals away from simple calisthenics and focusing on broader multimodal approaches like yoga.

## Data Availability

The original contributions presented in the study are included in the article/**Supplementary Material**, further inquiries can be directed to the corresponding author.

## Supplementary material

Supplement A to Yoga and Pain: a Mind-Body Complex System

Deepak Chopra^1†^, Eddie Stern^2^, William C Bushell^3^, Ryan D Castle^4†^
