# Supplementary material for: Yoga and pain: A mind-body complex system
Source: Front Pain Res (Lausanne). 2023 Feb 23;4:1075866. doi: 10.3389/fpain.2023.1075866 (PMC9996306; doi:10.3389/fpain.2023.1075866)
Supplement: Supplementary file 1 [file Datasheet1.pdf]

Supplement A to

Yoga and Pain: a Mind-Body Complex System

Deepak Chopra[1†], Eddie Stern[2], William C Bushell[3], Ryan D Castle[4†]

Network analysis results

Betweenness

Betweenness is a measure of centrality in a graph based on shortest paths. For every pair of node in a connected network, there exists at least one shortest path between the vertices such that the number of edges that the path passes through.[i] The betweenness for each node is the number of these shortest paths that pass through the node. Betweenness represents the degree to which nodes stand between each other. While other metrics identify nodes that have the greatest input or output, betweenness helps identify the most heavily trafficked pathways.

As seen in Table 1, the nodes with the highest betweenness tended to be STUDIES, with the highest values occurring in nodes about complex systems, followed closely by nodes related to pain pathways and pain management. There are a small number of nodes related to yoga or mind-body interventions No KEYWORDS were in the top 30.

Table 1

Label	Type	Metric	Value
**Small Open Chemical Systems Theory and Its Implications to Darwinian Evolutionary Dynamics, Complex Self-Organization and Beyond**	**Study**	betweenness	1.76E-05
**Extreme value theory of evolving phenomena in complex dynamical systems: firing cascades in a model of neural network**	**Study**	betweenness	1.66E-05
**Understanding and Modelling the Complexity of the Immune System: Systems Biology for Integration and Dynamical Reconstruction of Lymphocyte Multi-Scale Dynamics**	**Study**	betweenness	1.54E-05
**Complexity of Model Testing for Dynamical Systems with Toric Steady States**	**Study**	betweenness	1.5E-05
**Designer dynamics through chaotic traps: Controlling complex behavior in driven nonlinear systems**	**Study**	betweenness	7.55E-06
**Life as Complex Systems --- Viewpoint from Intra-Inter Dynamics**	**Study**	betweenness	6.14E-06
**Angiotensin II Triggers Peripheral Macrophage-to-Sensory Neuron Redox Crosstalk to Elicit Pain**	**Study**	betweenness	5.35E-06
Common Brain Mechanisms of Chronic Pain and Addiction	**Study**	betweenness	4.44E-06
Immediate preoperative outcomes of pain neuroscience education for patients undergoing total knee arthroplasty: A case series	**Study**	betweenness	4.32E-06
**Applying Complexity Theory to a Dynamical Process Model of the Development of Pathological Belief Systems**	**Study**	betweenness	4.18E-06
**The evolution of self-control**	**Study**	betweenness	4E-06
Low- Versus High-Intensity Plyometric Exercise During Rehabilitation After Anterior Cruciate Ligament Reconstruction	**Study**	betweenness	3.95E-06
**Forecasting transitions in systems with high dimensional stochastic complex dynamics: A Linear Stability Analysis of the Tangled Nature Model**	**Study**	betweenness	3.58E-06
A Mechanism-Based Approach to the Management of Osteoarthritis Pain	**Study**	betweenness	3.53E-06
Influence of a periodized circuit training protocol on intermuscular adipose tissue of patients with knee osteoarthritis: protocol for a randomized controlled trial	**Study**	betweenness	3.33E-06
Psychological processing in chronic pain: a neural systems approach	**Study**	betweenness	3.21E-06
**Corticotrophin-releasing factor 1 activation in the central amygdale and visceral hyperalgesia**	**Study**	betweenness	3.2E-06
**Cultural adaptation framework of social interventions in mental health: Evidence-based case studies from low- and middle-income countries**	**Study**	betweenness	3.17E-06
**Elite competitive swimmers exhibit higher motor cortical inhibition and superior sensorimotor skills in a water environment**	**Study**	betweenness	3.13E-06

**Remote ischemic conditioning as a cytoprotective strategy in vasculopathies during hyperhomocysteinemia: An emerging research perspective**	**Study**	betweenness	3.12E-06
Assessing for unique immunomodulatory and neuroplastic profiles of physical activity subtypes: a focus on psychiatric disorders	**Study**	betweenness	3.12E-06
**Impact of complex, partially nested clustering in a three-arm individually randomized group treatment trial: A case study with the wHOPE trial**	**Study**	betweenness	3.06E-06
**Changes of meningeal excitability mediated by corticotrigeminal networks: a link for the endogenous modulation of migraine pain**	**Study**	betweenness	3.04E-06
**A Dynamical Similarity Approach to the Foundations of Complexity and Coordination in Multiscale Systems**	**Study**	betweenness	2.87E-06
**Creating Inclusive Physical Activity Spaces: The Case of Body-Positive Yoga**	**Study**	betweenness	2.86E-06
**Using focus group methods to develop multicultural cancer pain education materials**	**Study**	betweenness	2.77E-06
**A Randomized, Single-Blind Study Evaluating the Effect of a Bone Pain Education Video on Reported Bone Pain in Patients with Breast Cancer Receiving Chemotherapy and Pegfilgrastim**	**Study**	betweenness	2.75E-06
**A dynamical model of fast cortical reorganization**	**Study**	betweenness	2.74E-06
**General Pathways of Pain Sensation and the Major Neurotransmitters Involved in Pain Regulation**	**Study**	betweenness	2.74E-06
Complex System	**Topics**	betweenness	0.000119

Table 1

Closeness

The value of closeness centrality, or closeness, determines the distance each vertex is from every other vertex. Points with high closeness tend to be highly correlated with the trends of the broader network. In this study closeness reflects many of the same patterns as degree centrality with slight variations due to interpreting limited quality from its connections, meaning connection to other highly connected vertices increases the value. This indicates a highly interdependent part of the network.

As seen in Table 2, the nodes with the largest closeness values were TOPICS, especially related to pain pathways, yoga interventions, and inflammation. Complex systems ranked relatively low as a TOPIC. STUDIES were significantly lower in value and were led by nodes related to pain pathways, inflammation, sensitization/catastrophization, and mind-body treatments. No KEYWORDS were present in the top 30.

Table 2

Label	Type	Metric	Value
**Angiotensin II Triggers Peripheral Macrophage-to-Sensory Neuron Redox Crosstalk to Elicit Pain**	**Study**	closeness	0.01494
Psychological processing in chronic pain: a neural systems approach	**Study**	closeness	0.014458
Immediate preoperative outcomes of pain neuroscience education for patients undergoing total knee arthroplasty: A case series	**Study**	closeness	0.013976
Influence of a periodized circuit training protocol on intermuscular adipose tissue of patients with knee osteoarthritis: protocol for a randomized controlled trial	**Study**	closeness	0.013976
**Biopsychosocial Influence on Shoulder Pain: Influence of Genetic and Psychological Combinations on Twelve-Month Postoperative Pain and Disability Outcomes**	**Study**	closeness	0.013494
Common Brain Mechanisms of Chronic Pain and Addiction	**Study**	closeness	0.01253
Assessing for unique immunomodulatory and neuroplastic profiles of physical activity subtypes: a focus on psychiatric disorders	**Study**	closeness	0.012048
Low- Versus High-Intensity Plyometric Exercise During Rehabilitation After Anterior Cruciate Ligament Reconstruction	**Study**	closeness	0.012048
A Mechanism-Based Approach to the Management of Osteoarthritis Pain	**Study**	closeness	0.011566
**Disease-Related, Nondisease-Related, and Situational Catastrophizing in Sickle Cell Disease and Its Relationship With Pain**	**Study**	closeness	0.011566
**Mind-body therapies and control of inflammatory biology: A descriptive review**	**Study**	closeness	0.011566
**Pain, psychosocial tests, pain sensitization and laparoscopic pelvic surgery**	**Study**	closeness	0.011566
**Pilot study of inflammatory responses following a negative imaginal focus in persons with chronic pain: analysis by sex/gender**	**Study**	closeness	0.011566

**Biopsychosocial influence on shoulder pain: Rationale and protocol for a pre-clinical trial**	**Study**	closeness	0.011084
**Generalized Pain Sensitization and Endogenous Oxytocin in Individuals With Symptoms of Migraine: A Cross-Sectional Study**	**Study**	closeness	0.011084
**Inflammation-induced pain sensitization in men and women: does sex matter in experimental endotoxemia?**	**Study**	closeness	0.011084
**Mindfulness-based stress reduction in relation to quality of life, mood, symptoms of stress, and immune parameters in breast and prostate cancer outpatients**	**Study**	closeness	0.011084
**Pain Catastrophizing and Quality of Life in Adults With Chronic Rhinosinusitis**	**Study**	closeness	0.011084
**Painful After-Sensations in Fibromyalgia are Linked to Catastrophizing and Differences in Brain Response in the Medial Temporal Lobe**	**Study**	closeness	0.011084
**Benefits of Yoga on IL-6: Findings from a Randomized Controlled Trial of Yoga for Depression**	**Study**	closeness	0.010602
**Intensive virtual reality and robotic based upper limb training compared to usual care, and associated cortical reorganization, in the acute and early sub-acute periods post-stroke: a feasibility study**	**Study**	closeness	0.010602
**Self-help Cognitive Behavioral Therapy Improves Health-Related Quality of Life for Inflammatory Bowel Disease Patients: A Randomized Controlled Effectiveness Trial**	**Study**	closeness	0.010602
**The effect of threat information on acquisition, extinction, and reinstatement of experimentally conditioned fear of movement-related pain**	**Study**	closeness	0.010602
PAIN RESPONSES	**Topics**	closeness	0.183614
Yoga Efficacy	**Topics**	closeness	0.167711
Pain & Inflammation	**Topics**	closeness	0.141446
Yoga & Pain Response	**Topics**	closeness	0.109398
**Rewriting Pain**	**Topics**	closeness	0.076145
**Yoga Cultural**	**Topics**	closeness	0.064578
**Complex Systems & Pain**	**Topics**	closeness	0.014578

Table 2

Degree centrality & indegree

The measurement of degree centrality, or degree, involves a basic, undirected count of the total connections linked to a vertex. It is solely based in quantity; the quality of connections does not affect the value. Degree centrality can be useful for identifying popular connectors or local hubs, but it does not necessarily reflect the behavior of the broader network. For the purposes of this study, degree centrality generally tracks studies which the largest number of relevant keywords. Indegree is a submetric of degree centrality that exclusively measures a node's incoming connections. Nodes that have disproportionate incoming connections tend to be destinations for information or have an output outside of the network.

As seen in Table 3, KEYWORDS were the largest group in degree centrality and the only group for indegree metrics. For both groups demographic identifiers were the highest ranking, followed by nodes related to yoga, pain, and pain pathways. Pain responses and yoga's efficacy were the highest ranking TOPICS. Only one STUDY was in the top 30, related to neurological measurements of pain.

Table 3

Label	Type	Metric	Value
HUMANS	**Keyword**	degree	408
FEMALE	**Keyword**	degree	181
MALE	**Keyword**	degree	149
ADULT	**Keyword**	degree	143
YOGA	**Keyword**	degree	127
MIDDLE AGED	**Keyword**	degree	120
**PAIN**	**Keyword**	degree	98
AGED	**Keyword**	degree	65

PAIN MEASUREMENT	Keyword	degree	65
QUALITY OF LIFE	Keyword	degree	65
CHRONIC PAIN	Keyword	degree	58
YOUNG ADULT	Keyword	degree	54
CATASTROPHIZATION	Keyword	degree	46
MEDITATION	Keyword	degree	45
RANDOMIZED CONTROLLED TRIALS AS TOPIC	Keyword	degree	45
TREATMENT OUTCOME	Keyword	degree	43
DEPRESSION	Keyword	degree	42
SURVEYS AND QUESTIONNAIRES	Keyword	degree	41
PAIN THRESHOLD	Keyword	degree	39
ADOLESCENT	Keyword	degree	37
INFLAMMATION	Keyword	degree	37
CROSS-SECTIONAL STUDIES	Keyword	degree	35
ANXIETY	Keyword	degree	34
CENTRAL NERVOUS SYSTEM SENSITIZATION	Keyword	degree	33
EXERCISE	Keyword	degree	31
Angiotensin II Triggers Peripheral Macrophage-to-Sensory Neuron Redox Crosstalk to Elicit Pain	Study	degree	32
PAIN RESPONSES	Topics	degree	113
Yoga Efficacy	Topics	degree	110
Pain & Inflammation	Topics	degree	74
Yoga & Pain Response	Topics	degree	56

Label	Type	Metric	Value
HUMANS	Keyword	indegree	408
FEMALE	Keyword	indegree	181
MALE	Keyword	indegree	149
ADULT	Keyword	indegree	143
YOGA	Keyword	indegree	127
MIDDLE AGED	Keyword	indegree	120
PAIN	Keyword	indegree	98
AGED	Keyword	indegree	65
PAIN MEASUREMENT	Keyword	indegree	65
QUALITY OF LIFE	Keyword	indegree	65
CHRONIC PAIN	Keyword	indegree	58
YOUNG ADULT	Keyword	indegree	54
CATASTROPHIZATION	Keyword	indegree	46
MEDITATION	Keyword	indegree	45
RANDOMIZED CONTROLLED TRIALS AS TOPIC	Keyword	indegree	45
TREATMENT OUTCOME	Keyword	indegree	43
DEPRESSION	Keyword	indegree	42
SURVEYS AND QUESTIONNAIRES	Keyword	indegree	41
PAIN THRESHOLD	Keyword	indegree	39
ADOLESCENT	Keyword	indegree	37
INFLAMMATION	Keyword	indegree	37
CROSS-SECTIONAL STUDIES	Keyword	indegree	35
ANXIETY	Keyword	indegree	34
CENTRAL NERVOUS SYSTEM SENSITIZATION	Keyword	indegree	33
COMPLEMENTARY THERAPIES	Keyword	indegree	31

EXERCISE	**Keyword**	indegree	31
**ANIMALS**	**Keyword**	indegree	30
**MINDFULNESS**	**Keyword**	indegree	24
**EXERCISE THERAPY**	**Keyword**	indegree	23
**STRESS, PSYCHOLOGICAL**	**Keyword**	indegree	23

Table 3

Eigenvector

Where degree centrality strictly measures quantity, and closeness centrality measures quantity with a small influence of quality, eigenvector centrality emphasizes quality of connection over quantity. Eigenvector values measure how well connected any given vertex is to the other most well-connected vertices. In general vertices with high eigenvector values reflect the leading edge of a network. Though they may not be as widely connected as other values, they tend to have disproportionate influence on the system. In the context of this study, eigenvector centrality is associated with nonlinear connections between areas of study.

As seen in Table 4, STUDIES had some of the highest value eigenvector nodes, and were nearly all related to biological complex systems. KEYWORDS were the largest block of high ranking eigenvector nodes, with all the top nodes being related to complex systems and modeling. Immune and inflammatory systems were next, followed by nodes related to pain, then several related to biology and physics. There was one TOPIC node, referencing complex systems.

Table 4

Label	Type	Metric	Value
**MODELING, COMPLEX SYSTEM**	**Keyword**	eigenvector	0.030769
**QUANTITATIVE BIOLOGY - QUANTITATIVE METHODS**	**Keyword**	eigenvector	0.030769
**BIOLOGICAL SYSTEM**	**Keyword**	eigenvector	0.023077
**MODELING, BIOLOGICAL**	**Keyword**	eigenvector	0.023077
**NONLINEAR SCIENCES - ADAPTATION AND SELF-ORGANIZING SYSTEMS**	**Keyword**	eigenvector	0.023077
**QUANTITATIVE BIOLOGY - NEURONS AND COGNITION**	**Keyword**	eigenvector	0.023077
**COMPLEX ADAPTIVE SYSTEM**	**Keyword**	eigenvector	0.015385
**NONLINEAR SCIENCES - CHAOTIC DYNAMICS**	**Keyword**	eigenvector	0.015385
**COMPUTER SCIENCE - SYMBOLIC COMPUTATION**	**Keyword**	eigenvector	0.007692
**IMMUNE SYSTEM**	**Keyword**	eigenvector	0.007692
**INFLAMMATION**	**Keyword**	eigenvector	0.007692
**MATHEMATICS - ALGEBRAIC GEOMETRY**	**Keyword**	eigenvector	0.007692
**MATHEMATICS - DYNAMICAL SYSTEMS**	**Keyword**	eigenvector	0.007692
**PAIN**	**Keyword**	eigenvector	0.007692
**PAIN RESPONSE**	**Keyword**	eigenvector	0.007692
**PHYSICS - BIOLOGICAL PHYSICS**	**Keyword**	eigenvector	0.007692
**PHYSICS - CHEMICAL PHYSICS**	**Keyword**	eigenvector	0.007692
**QUANTITATIVE BIOLOGY**	**Keyword**	eigenvector	0.007692
**QUANTITATIVE BIOLOGY - CELL BEHAVIOR**	**Keyword**	eigenvector	0.007692
**QUANTITATIVE BIOLOGY - TISSUES AND ORGANS**	**Keyword**	eigenvector	0.007692
**A Dynamical Similarity Approach to the Foundations of Complexity and Coordination in Multiscale Systems**	**Study**	eigenvector	0.069231
**Applying Complexity Theory to a Dynamical Process Model of the Development of Pathological Belief Systems**	**Study**	eigenvector	0.069231
**Complexity of Model Testing for Dynamical Systems with Toric Steady States**	**Study**	eigenvector	0.069231

**Designer dynamics through chaotic traps: Controlling complex behavior in driven nonlinear systems**	**Study**	eigenvector	0.069231
**Extreme value theory of evolving phenomena in complex dynamical systems: firing cascades in a model of neural network**	**Study**	eigenvector	0.069231
**Forecasting transitions in systems with high dimensional stochastic complex dynamics: A Linear Stability Analysis of the Tangled Nature Model**	**Study**	eigenvector	0.069231
**Life as Complex Systems --- Viewpoint from Intra-Inter Dynamics**	**Study**	eigenvector	0.069231
**Small Open Chemical Systems Theory and Its Implications to Darwinian Evolutionary Dynamics, Complex Self-Organization and Beyond**	**Study**	eigenvector	0.069231
**Understanding and Modelling the Complexity of the Immune System: Systems Biology for Integration and Dynamical Reconstruction of Lymphocyte Multi-Scale Dynamics**	**Study**	eigenvector	0.069231
Complex System	**Topics**	eigenvector	0.069231

Table 4

Reach efficiency

Reach measures the portion of the network within two steps of an element. In general, elements with high reach can spread information through the network through close friend-of-a-friend contacts. Reach efficiency normalizes reach by dividing it by size (number of neighbors). In general, elements with high reach efficiency are less connected but gain more exposure through each direct relationship. Reach efficiency is useful for determining influence as well as indicating how coherent and consistent that influence is.

As seen in Table 5, reach efficiency was mostly split between KEYWORDS and STUDIES in terms of quantity, but the highest values were among STUDIES. The leading STUDY nodes all involved complex systems and pain. The leading KEYWORDS were highly heterogeneous and patterns were not readily identifiable.

Table 5

**Label**	**Type**	**Metric**	**Value**
**ACCIDENTS, HOME**	**Keyword**	reach-efficiency	0.000241
**ACQUIRED BRAIN INJURY**	**Keyword**	reach-efficiency	0.000241
**ANALYSIS OF VARIANCE**	**Keyword**	reach-efficiency	0.000241
**ANTERIOR CINGULATE CORTEX**	**Keyword**	reach-efficiency	0.000241
**CANCER RELATED PAIN**	**Keyword**	reach-efficiency	0.000241
**CELLS, CULTURED**	**Keyword**	reach-efficiency	0.000241
**COUNSELING**	**Keyword**	reach-efficiency	0.000241
**GASTROESOPHAGEAL REFLUX**	**Keyword**	reach-efficiency	0.000241
**LASERS**	**Keyword**	reach-efficiency	0.000241
**LEADERSHIP**	**Keyword**	reach-efficiency	0.000241
**PSYCHOLOGICAL TREATMENT**	**Keyword**	reach-efficiency	0.000241
**REPETITIVE SENSORY STIMULATION**	**Keyword**	reach-efficiency	0.000241
**SUPINE POSITION**	**Keyword**	reach-efficiency	0.000241
**A Dynamical Similarity Approach to the Foundations of Complexity and Coordination in Multiscale Systems**	**Study**	reach-efficiency	0.001252
**COMPLEX SYSTEMS**	**Study**	reach-efficiency	0.001252
**Forecasting transitions in systems with high dimensional stochastic complex dynamics: A Linear Stability Analysis of the Tangled Nature Model**	**Study**	reach-efficiency	0.001252
**Life as Complex Systems --- Viewpoint from Intra-Inter Dynamics**	**Study**	reach-efficiency	0.001252
**Applying Complexity Theory to a Dynamical Process Model of the Development of Pathological Belief Systems**	**Study**	reach-efficiency	0.001124
**Designer dynamics through chaotic traps: Controlling complex behavior in driven nonlinear systems**	**Study**	reach-efficiency	0.001124
**Complexity of Model Testing for Dynamical Systems with Toric Steady States**	**Study**	reach-efficiency	0.001032

**CRPS: A contingent hypothesis with prostaglandins as crucial conversion factor**	**Study**	reach-efficiency	0.001032
**Small Open Chemical Systems Theory and Its Implications to Darwinian Evolutionary Dynamics, Complex Self-Organization and Beyond**	**Study**	reach-efficiency	0.001032
**Extreme value theory of evolving phenomena in complex dynamical systems: firing cascades in a model of neural network**	**Study**	reach-efficiency	0.000963
**Structure and dynamics of dynorphin peptide and its receptor**	**Study**	reach-efficiency	0.000963
**Painful intelligence: What AI can tell us about human suffering**	**Study**	reach-efficiency	0.00091
**Understanding and Modelling the Complexity of the Immune System: Systems Biology for Integration and Dynamical Reconstruction of Lymphocyte Multi-Scale Dynamics**	**Study**	reach-efficiency	0.00091
**Hormesis, adaptation, and the sandpile model**	**Study**	reach-efficiency	0.000867
**Pain pathogenesis in rheumatoid arthritis -- what have we learned from animal models**	**Study**	reach-efficiency	0.000867
**A Deep Learning Approach to Diagnosing Multiple Sclerosis from Smartphone Data**	**Study**	reach-efficiency	0.000803
**Complex adaptive systems allostasis in fibromyalgia**	**Study**	reach-efficiency	0.000803

Table 5

Synthesis

All nodes (studies, topics, and keywords) were mapped through the network analysis and the highest 30 values for each network metric isolated. Every node was cross-referenced and any node that had multiple high network metric values were highlighted for examination. Leaders in this synthesis will be helpful in identifying the similarities in keywords and patterns of topics between fields that are not usually linked.

As seen in Table 6, several nodes were high ranking across three metrics, potentially acting as indicators of the broader pattern of the research.

Table 6

**Label**	**Type**
**A Dynamical Similarity Approach to the Foundations of Complexity and Coordination in Multiscale Systems**	Study
**Angiotensin II Triggers Peripheral Macrophage-to-Sensory Neuron Redox Crosstalk to Elicit Pain**	Study
**Applying Complexity Theory to a Dynamical Process Model of the Development of Pathological Belief Systems**	Study
**Complexity of Model Testing for Dynamical Systems with Toric Steady States**	Study
**Designer dynamics through chaotic traps: Controlling complex behavior in driven nonlinear systems**	Study
**Extreme value theory of evolving phenomena in complex dynamical systems: firing cascades in a model of neural network**	Study
**Forecasting transitions in systems with high dimensional stochastic complex dynamics: A Linear Stability Analysis of the Tangled Nature Model**	Study
**INFLAMMATION**	Keyword
**Life as Complex Systems --- Viewpoint from Intra-Inter Dynamics**	Study
**PAIN**	Keyword
**Small Open Chemical Systems Theory and Its Implications to Darwinian Evolutionary Dynamics, Complex Self-Organization and Beyond**	Study
**Understanding and Modelling the Complexity of the Immune System: Systems Biology for Integration and Dynamical Reconstruction of Lymphocyte Multi-Scale Dynamics**	Study

Table 6: (Ryan Castle)

The majority of the most influential nodes were STUDIES relating to complex systems and nodes relating to pain pathways. This suggests there are significant intersections between the study of complex systems and pain.

As seen in Table 7, a larger number of nodes were leaders among two metrics, and could be considered part of a larger, defining pattern between the fields of pain management and mind-body therapies.

Table 7

Label	Type
A Mechanism-Based Approach to the Management of Osteoarthritis Pain	Study
ADOLESCENT	Keyword
ADULT	Keyword
AGED	Keyword
ANXIETY	Keyword
Assessing for unique immunomodulatory and neuroplastic profiles of physical activity subtypes: a focus on psychiatric disorders	Study
CATASTROPHIZATION	Keyword
CENTRAL NERVOUS SYSTEM SENSITIZATION	Keyword
CHRONIC PAIN	Keyword
Common Brain Mechanisms of Chronic Pain and Addiction	Study
Complex System	Topics
CROSS-SECTIONAL STUDIES	Keyword
DEPRESSION	Keyword
EXERCISE	Keyword
FEMALE	Keyword
HUMANS	Keyword
Immediate preoperative outcomes of pain neuroscience education for patients undergoing total knee arthroplasty: A case series	Study
Influence of a periodized circuit training protocol on intermuscular adipose tissue of patients with knee osteoarthritis: protocol for a randomized controlled trial	Study
Low- Versus High-Intensity Plyometric Exercise During Rehabilitation After Anterior Cruciate Ligament Reconstruction	Study
MALE	Keyword
MEDITATION	Keyword
MIDDLE AGED	Keyword
Pain & Inflammation	Topics
PAIN MEASUREMENT	Keyword
PAIN RESPONSES	Topics
PAIN THRESHOLD	Keyword
Psychological processing in chronic pain: a neural systems approach	Study
QUALITY OF LIFE	Keyword
RANDOMIZED CONTROLLED TRIALS AS TOPIC	Keyword
SURVEYS AND QUESTIONNAIRES	Keyword
TREATMENT OUTCOME	Keyword
YOGA	Keyword
Yoga & Pain Response	Topics
Yoga Efficacy	Topics
YOUNG ADULT	Keyword

Table 7

Setting aside demographic identifier KEYWORDS, the nodes that were leading in two separate metrics tend to involve pain management, chronic pain, neuroscience, pain education, sensitization/catastrophization, and yoga or meditation. This suggests that the broader literature review highlights the close connections between chronic pain and mind-body therapies.

Combining the blocks of synthesized findings provides evidence that the fields of study regarding complex systems, pain management, and mind-body therapies share many of the same topics, keywords, and published studies. The literature review suggests the fields share significant patterns.

[i] Freeman, L. C. (1977). A Set of Measures of Centrality Based on Betweenness. Sociometry, 40(1), 35–41. https://doi.org/10.2307/3033543
